# Endocrine Pancreas Development and Dysfunction Through the Lens of Single-Cell RNA-Sequencing

**DOI:** 10.3389/fcell.2021.629212

**Published:** 2021-04-29

**Authors:** Wojciech J. Szlachcic, Natalia Ziojla, Dorota K. Kizewska, Marcelina Kempa, Malgorzata Borowiak

**Affiliations:** ^1^Department of Gene Expression, Institute of Molecular Biology and Biotechnology, Faculty of Biology, Adam Mickiewicz University, Poznań, Poland; ^2^Department of Molecular and Cellular Biology, Baylor College of Medicine, Houston, TX, United States

**Keywords:** single-cell RNA sequencing, pancreas development, stem cell pancreatic differentiation, beta cell development and maturation, diabetes

## Abstract

A chronic inability to maintain blood glucose homeostasis leads to diabetes, which can damage multiple organs. The pancreatic islets regulate blood glucose levels through the coordinated action of islet cell-secreted hormones, with the insulin released by β-cells playing a crucial role in this process. Diabetes is caused by insufficient insulin secretion due to β-cell loss, or a pancreatic dysfunction. The restoration of a functional β-cell mass might, therefore, offer a cure. To this end, major efforts are underway to generate human β-cells *de novo, in vitro*, or *in vivo*. The efficient generation of functional β-cells requires a comprehensive knowledge of pancreas development, including the mechanisms driving cell fate decisions or endocrine cell maturation. Rapid progress in single-cell RNA sequencing (scRNA-Seq) technologies has brought a new dimension to pancreas development research. These methods can capture the transcriptomes of thousands of individual cells, including rare cell types, subtypes, and transient states. With such massive datasets, it is possible to infer the developmental trajectories of cell transitions and gene regulatory pathways. Here, we summarize recent advances in our understanding of endocrine pancreas development and function from scRNA-Seq studies on developing and adult pancreas and human endocrine differentiation models. We also discuss recent scRNA-Seq findings for the pathological pancreas in diabetes, and their implications for better treatment.

## Pancreas and Diabetes

The pancreas is a glandular organ with crucial roles in digestion (exocrine activity) and glucose homeostasis (endocrine activity). The main mass of the exocrine compartment consists of the *acini.* The acinar cells secrete enzymes involved in the digestion of proteins (trypsinogen, chymotrypsinogen), fats (lipase, phospholipase, cholesterol esterase), and carbohydrates (amylase). These enzymes are produced in an inactive form, stored as granules known as zymogens, and are released into the ducts as required; these ducts eventually converge into the main duct, via which the pancreatic juice drains directly into the duodenum (Alexandre-Heymann et al., 2019).

The exocrine cells occupy a large proportion of the pancreas, whereas the endocrine compartment consists of cell clusters known as the islets of Langerhans (about 100 μm in diameter) dispersed throughout the organ. Crosstalk between the different cell types comprising the islets (α-, β-, δ-, ε-, and PP-cells) regulates glucose homeostasis, by controlling the secretion of cell-specific hormones (Benitez et al., 2012). The most abundant cells in the pancreatic islets (up to 90% per islet) are the α- and β-cells. The β-cells secrete insulin in response to an increase in serum glucose concentration. Insulin facilitates the uptake of glucose from the blood by cells in peripheral tissues, for use as an energy source (in muscles), or storage as glycogen (in hepatocytes) and thus the insulin decreases serum glucose levels. By contrast, the α-cells secrete glucagon, which increases blood sugar levels by stimulating the release of glucose from glycogen in hepatocytes (Benitez et al., 2012). The hormones secreted by the other islet cells regulate the function of α- and β-cells, and their own functions, in a paracrine manner. The δ-cells secrete somatostatin, which regulates the functions of both α- and β-cells by binding to the somatostatin receptors expressed on their membranes (Braun et al., 2010; Benitez et al., 2012; Gromada et al., 2018). The PP-cells produce and release pancreatic polypeptide, which reduces the secretion of glucagon from α-cells (Aragón et al., 2015). The ε-cells, a rare population within the islet (<1% in adult human islets), release ghrelin to stimulate somatostatin secretion and inhibit insulin secretion (Dominguez Gutierrez et al., 2018). Insufficient paracrine regulation within the islets may lead to the long-term disruption of glucose balance, resulting in a chronic metabolic disease, diabetes mellitus.

Diabetes is considered a disease of civilization. It affected over 425 million people worldwide in 2017, and the number of people with diabetes is predicted to increase to 642 million by 2040 (idf.org, Velazco-Cruz et al., 2019). The various types of diabetes differ in etiology and molecular background. However, the hallmark of this disease is insufficient insulin secretion due to β-cell loss resulting from autoimmune attack, as in type 1 diabetes (T1D), or an impairment of the β-cell function or insulin resistance built up by peripheral tissues, as in type 2 diabetes (T2D) (American Diabetes and Association, 2013; Johannesson et al., 2015). Most patients with diabetes have type 1 or type 2 disease, but there is also another rare type of diabetes (accounting for 1–2% cases): maturity-onset diabetes of the young (MODY), which is caused by a single gene mutation. MODY patients are usually diagnosed in late childhood or early adulthood, have a strong family history of diabetes, lack autoantibodies against pancreatic antigens, and have normal body weight. MODY manifests as various abnormalities of β-cell function and can be further classified into subtypes on the basis of the gene mutated (Gardner and Tai, 2012). Many of the causal genes for MODY encode factors involved in pancreas development, suggesting that this form of diabetes may be a developmental disease.

Current therapeutic strategies for diabetes aim to control carbohydrate homeostasis, mostly through the delivery of exogenous insulin, the use of drugs to stimulate insulin production and secretion by the diminishing β-cell mass, and therapeutic agents inhibiting glucagon secretion (Marín-Peñalver et al., 2016). A healthy lifestyle, including an appropriate diet and physical activity, is also promoted in addition to pharmacological treatment. These approaches might stabilize blood glucose levels but are often imprecise and burdensome for patients. For example, insulin treatment requires multiple injections per day of an exact amount of insulin (Tan et al., 2019), making it challenging to maintain correct glucose homeostasis over long periods. Therefore, patients with diabetes often develop secondary diseases affecting multiple organs, including cardiovascular or renal functions, which may reduce their lifespan and quality of life. There is, thus, a pressing need to identify a cure for diabetes, or better treatments, to free patients from the inconvenience and risks associated with this highly prevalent disease.

One attractive idea is the restoration of a functional β-cell pool in the patient (Zhou and Melton, 2018). Proof-of-principle studies have shown that islet transplantation can lead to independence from exogenous insulin in people with diabetes (Bellin et al., 2012; Moore et al., 2015). Ready-to-transplant islets containing insulin-secreting cells are obtained from cadaveric donors. However, this source of cells is limited, due to a shortage of donors and suboptimal islet isolation procedures (Shapiro et al., 2006; Bellin et al., 2012). Xenotransplantation with immunocompetent porcine islets, a potentially unlimited source of β-cells, has been proposed as a means of solving this problem. However, the risk of transmitting endogenous porcine retroviruses to patients and the high inflammatory response to animal islets have prevented the transfer of this approach into clinical practice (van der Windt et al., 2012; Rashid et al., 2014; Moore et al., 2015).

Recent advances in regenerative medicine based on the use of β-cells differentiated *in vitro* from pluripotent stem cells (PSCs) have raised the possibility of an inexhaustible source of β-cells (Johannesson et al., 2015). Application of the knowledge obtained in classical biology studies, including knockout or lineage tracing experiment has led to the *de novo* derivation of β-cells, resulting in improvements in glucose homeostasis in diabetic mice (Pagliuca et al., 2014; Rezania et al., 2014; Russ et al., 2015; Nair et al., 2019).

Moreover, in addition to their possible use in cell replacement therapies for diabetes, PSC-derived β-cells could be used as a platform for disease modeling and drug screening. The *in vivo* transdifferentiation of other cell types into β-cells has also been proposed as an alternative to transplantation. In mouse T1D models, almost complete β-cell loss triggered the transdifferentiation of α- or δ-cells into insulin-producing cells (Thorel et al., 2010; Bru-Tari et al., 2019). Furthermore, pancreatic acinar cells and endocrine cells in the intestine or stomach can be transdifferentiated into insulin-secreting cells (Zhou et al., 2008; Li et al., 2014; Ariyachet et al., 2016). However, the transdifferentiation efficiency is low, the long-term stability of newly formed β-cells is uncertain, and our understanding of this process remains incomplete (Collombat et al., 2009; Courtney et al., 2013; Wilcox et al., 2013; Chera et al., 2014; Li et al., 2017; Furuyama et al., 2019).

Significant progress has been made toward the efficient *in vitro* or *in vivo* production of clinically relevant, functional β-cells, but this aim has yet to be achieved. *In vitro* pancreatic differentiation or *in vivo* transdifferentiation require an understanding of pancreas development, derived from extensive and meticulous research on transgenic animal models and cell lines.

## Pancreas Development

The pancreas develops from the endoderm-derived primitive digestive tube. The gut tube separates into the foregut, midgut and hindgut, and pancreatic specification occurs in the duodenal loop, at the border between foregut and midgut (Wells and Melton, 2000; Lawson and Schoenwolf, 2003). Approximately 29–33 days post-conception (dpc) in humans, and at embryonic day 9.5–10 (E9.5-E10) in mice, the primary transition begins with gut tube budding, leading to the appearance of two pancreatic buds on the dorsal and ventral sides of the duodenal loop. The expression of pancreatic and duodenal homeobox 1 (PDX1) (Offield et al., 1996; Burlison et al., 2008), SRY-box transcription factor 9 (SOX9) (Lynn et al., 2007), and pancreas associated transcription factor 1A (PTF1A) (Krapp et al., 1998; Kawaguchi et al., 2002; Burlison et al., 2008) marks the multipotent pancreatic progenitors (MPs) within the buds. These MPs give rise to all pancreatic cell types. Subsequently, in the 6th week of human development and at E11-12 in mice, the buds bulge, and the ventral bud flips to the other side of the gut tube and fuses with the dorsal bud (Jeon et al., 2009). Progressive branching morphogenesis establishes the trunk (ductal body) and the tip (ductal termini) domains between the 10th and 14th weeks in humans, and at E13.5 in mice. This event marks the start of the so-called secondary transition in pancreatic development. The PTF1A^+^ and PDX1^+^ cells in the tip domain are initially multipotent, but they acquire an acinar fate bias during secondary transition (Zhou et al., 2007). The trunk domain contains PDX1^+^, SOX9^+^, and NKX6.1^+^, bipotent progenitors (BPs) that generate duct-like structures and endocrine progenitors (Jennings et al., 2015). The endocrine progenitors (EPs) arise from trunk domain cells lacking SOX9 and expressing neurogenin-3 (NEUROG3). The NEUROG3 transcription factor is necessary and sufficient for endocrine cell lineage specification (Gradwohl et al., 2000; Gu et al., 2002; Sheets et al., 2018) and activates downstream transcription factors essential for endocrine specification, such as neuronal differentiation 1 (NEUROD1), INSM transcriptional repressor 1 (INSM1), Iroquois homeobox 1 (IRX1), regulatory factor X6 (RFX6), and paired box 4 (PAX4). In mice, NEUROG3 is expressed in a biphasic manner. The first wave of NEUROG3 expression is associated with the emergence of pro-α-cells, from E8.5 to E11 (Larsson, 1998; Villasenor et al., 2008). The second wave of high NEUROG3 expression, from E13.5 to E17.5, leads to the generation of multiple endocrine cell types (Gradwohl et al., 2000; Schwitzgebel et al., 2000; Villasenor et al., 2008). In humans, NEUROG3 expression increases at 47–52 dpc, coinciding with the appearance of the first insulin-expressing cells, and peaks at 8–10 weeks of development (Jennings et al., 2013). All five types of pancreatic endocrine cells arise from NEUROG3^+^ EPs. A unique combination of transcription factors triggers islet cell type-specific gene regulatory networks in EPs and represses alternative networks, resulting in the formation of cells producing specific hormones.

## Single-Cell RNA Sequencing Technology

Tremendous efforts have been made to identify the principal transcription factors and signaling pathways driving pancreas development. However, conventional research methods may miss subtle molecular events, such as cell state transitions, and cellular heterogeneity, a knowledge of which is necessary for the fine-tuning of β-cell production and for understanding the molecular mechanisms underlying diabetes. These gaps in our knowledge can be bridged by the use of rapidly evolving single-cell transcriptomics (scRNA-Seq) and other single-cell omics technologies.

Global gene expression analysis by RNA-Seq became a very common technology in studies on pancreas development and disease. Yet, bulk gene expression analysis by RNA-Seq provides information about average levels of gene expression for all cells. Thus, with bulk RNA-Seq, it is almost impossible to detect continuous cell-state transitions, the cell fate bifurcations, transient molecular events, or rare cell types and subpopulations. In contrast, scRNA-Seq enables to define the transcriptome of individual cells within the studied tissue, organ, or in cell cultures. Cells are grouped into clusters based on transcriptomic similarities. The extraction of a cell type of interest from a larger dataset makes it possible to identify discrete cell subtypes of subpopulations, potentially reflecting subsequent also maturation steps. Individual cells can also be ordered in pseudotime, reflecting their putative sequential appearance, and aligned along linear developmental trajectories (Cannoodt et al., 2016; Qiu X. et al., 2017; Street et al., 2018; Tritschler et al., 2019; Van den Berge et al., 2020). Within these trajectories, it is possible to identify branching points for cell fate decisions. Moreover, the dynamics of cell state transitions can be inferred from RNA velocity (La Manno et al., 2018; Bergen et al., 2020). For each cell state or pseudotime point, transcripts, signaling pathways, and gene ontology terms displaying enrichment can be identified. A pseudodynamics method, in which whole cell populations are placed along pseudotime trajectories, was also proposed for the inference of developmental population dynamics from scRNA-Seq data (Fischer et al., 2019). Alternatively, a gene-centric approach can be used to align each gene expression over pseudotime, and cluster genes with similar patterns of behavior into regulons (Van de Sande et al., 2020). Each cell type can then be defined by its regulons. The strengths and limitations of pseudotime analyses have been recently discussed in detail elsewhere (Tritschler et al., 2019). Therefore, the principal advantage of scRNA-Seq is that it facilitates creation of novel scientific hypotheses, depending on the research goal, through a plethora of rapidly evolving bioinformatics tools (Efremova and Teichmann, 2020; Wu and Zhang, 2020). Yet, these hypotheses are based on a glimpse into complex biological systems, might be an artifact of bioinformatic analysis and have to be further validated by other methods.

**FIGURE 1:**
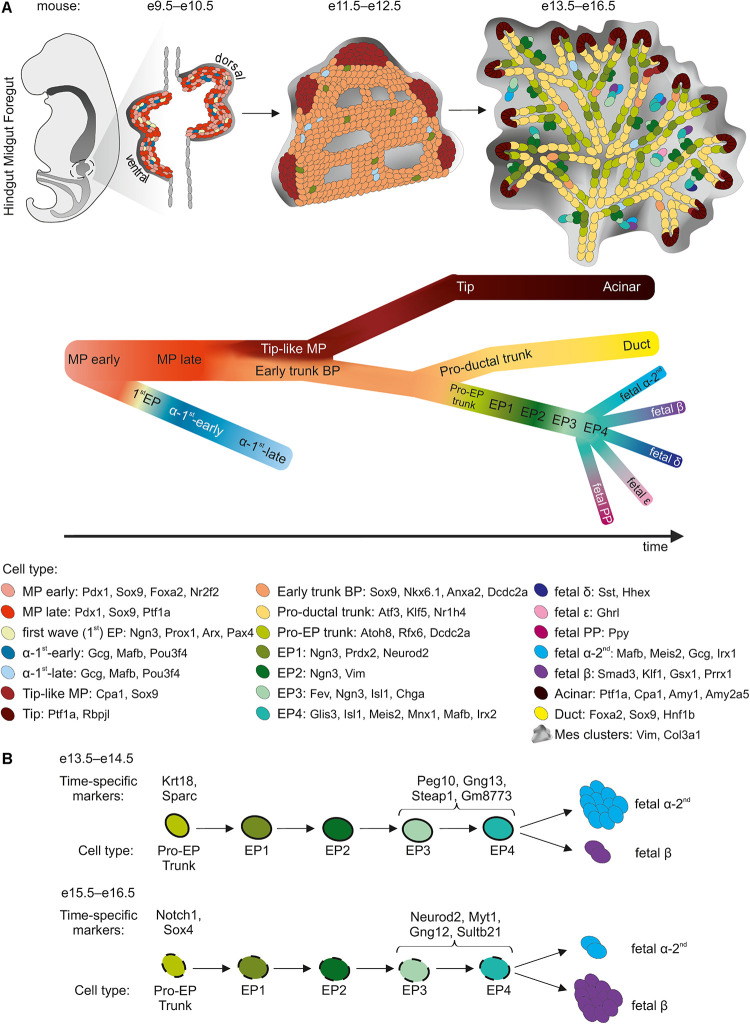
sc-RNA-Seq insight into murine pancreatic development. **(A)** Three major morphogenesis transitions in pancreatic development are shown. Cell types with cell specific markers are listed in the legend below. The black arrow indicates the developmental trajectory. MP, multipotent progenitors; BP, bipotent progenitors; EP, endocrine progenitors. **(B)** Developmental trajectory of Pro-EP trunk and EP cells as they differentiate at e13.5–e14.5 preferably into α-cells, and at e15.5–e16.5 preferably into β-cells. Time point-specific markers are listed above the scheme.

Since the first publication relating to scRNA-Seq in 2009 (Tang et al., 2009), single-cell techniques have rapidly evolved, and a multitude of sequencing platforms and bioinformatics tools for data analysis have been developed (Svensson et al., 2018). The platforms that initially dominated this sector, such as Smart-Seq (Ramsköld et al., 2012; Picelli et al., 2013), used flow-activated cell separation (FACS) to separate individual cells. A C1 Fluidigm microfluidics system was subsequently adapted to separate cells into reaction chambers on a chip. These low-throughput methods enabled to sequence hundreds of cells at a time and were relatively costly because of limited sample pooling. In more advanced platforms, such as the widely used Drop-seq (Macosko et al., 2015), inDrops (Klein et al., 2015), and 10× Genomics Chromium (Zheng et al., 2017), single cells are encapsulated in microfluidic drops. The mRNAs of individual cells are then tagged with a unique barcode sequence, allowing multiplexing. Droplet-based methods generally enable to sequence hundreds of thousands of cells in parallel and generate a number of reads similar to that for bulk RNA sequencing. However, the total read number must then be divided by the number of cells used in the experiment, and the use of larger numbers of cells results in a lower sequencing depth (Zhang et al., 2020). The detection limit for scRNA-Seq is, thus, much lower than that for bulk RNA-Seq (Mawla and Huising, 2019). Low-abundance RNAs therefore frequently remain undetected, due to technical noise, sequencing depth, or an actual biological effect (Lähnemann et al., 2020). Fewer than 100 transcripts are commonly detected in all β-cells, for example (Mawla and Huising, 2019). Nevertheless, a β-cell pool can be constituted based on a reasonable correlation with the corresponding bulk RNA-Seq data. The overrepresentation of highly abundant transcripts, such as that for insulin (INS) in the endocrine pancreas, for example, may also hinder the detection of less abundant transcripts. High-abundance transcripts might also be a source of biological contamination when free-floating mRNA is captured in a droplet (Macosko et al., 2015) and for example endocrine gene transcripts have been found to be abundant even in non-endocrine cells, probably due to contamination (Marquina-Sanchez et al., 2020). The use of cross-species spike-ins in samples can overcome this problem. The examples mentioned, and multiple other experimental and analytical limitations of scRNA-Seq have been discussed in detail elsewhere (Luecken and Theis, 2019; Mawla and Huising, 2019; Tritschler et al., 2019; Lähnemann et al., 2020). Despite these challenges, scRNA-Seq has already established itself as a valuable tool for developmental biology. A growing number of scRNA-Seq studies on endocrine pancreas development (summarized in Supplementary Table 1) have shown that at different developmental stages cell types, traditionally identified by a few markers, consist of heterogeneous cell populations reflecting continuous maturation or transitions between stages, and cell fate bias (Figure 1). Below, we will discuss examples of the corroboration and extension of our understanding of pancreas development by scRNA-Seq.

## Novel Insight Into Endocrine Pancreas Development From Single-Cell RNA-Seq

### Epithelial Multipotent Progenitors

Numerous knockout studies in mice have suggested that MPs expressing Pdx1, Sox9, and Ptf1a form a homogeneous population, in which each MP has a similar developmental potential. Recently, the scRNA-Seq of E9.5–E17.5 pancreas revealed the existence of three consecutive subpopulations of *Pdx1^+^ Sox9^+^* MPs: MP−early, MP−late, and tip-like cells (Yu et al., 2019). Previously undetected MP−early cells, expressing *Nr2f2* but not *Ptf1a*, were found at E9.5 and, by E10.5, were succeeded by a *Ptf1a^+^ Nr2f2^–^* MP-late population. Immunofluorescence staining confirmed the expression of NR2F2 in PDX1^+^ cells in the E9.5 pancreas, but not E10.5. The scRNA-Seq identify MP-early cells as a direct source of the first wave of *Neurog3*^+^ cells at E9.5, with these cells developing into α-cells (Yu et al., 2019). Consistent with this finding, lineage tracing and single-cell qPCR (sc-qPCR) showed that *Ptf1a*^+^ cells were rarely ancestors of the first *Neurog3*^+^ cells in the E9.5 pancreas (Larsen et al., 2017). Thus, the MP-early cells are probably the first pancreatic cells and serve as a branching point for cell fate decisions.

After MP expansion, branching morphogenesis begins at E12.5 driven by tip cells, self-renewing progenitors present at the ductal termini. Tip progenitors display stage-dependent multipotency (Zhou et al., 2007; Larsen et al., 2017; Sznurkowska et al., 2018). scRNA-Seq revealed that, by E11.5, the MP-late cluster gives rise to the tip-like progenitors expressing *Cpa1*, a marker of tip and acinar cells, and *Sox9*, a marker of the trunk and ducts (Yu et al., 2019). The *Cpa1*^+^ cells remain multipotent until E12.5-E13.5, yielding trunk and acinar cells, while from E13.5-E14.5 are restricted to the acinar lineage (Sznurkowska et al., 2018; Bastidas-Ponce et al., 2019; Yu et al., 2019). The identification of *Cpa1*^+^ multipotent tip cells in these scRNA-Seq experiments thus confirmed and extended a discovery made in an extensive *in situ* hybridization screen of over 1,100 transcription factors in mouse embryonic pancreas, followed by lineage tracing of Cpa1^+^ progeny (Zhou et al., 2007). Interestingly, a rare population of multipotent PDX1^+^/ALK3^+^/CAII- progenitors was recently identified by scRNA-Seq in adult human ducts (Qadir et al., 2020). Upon transplantation under the mouse kidney capsule, these progenitors differentiated into acinar, ductal, and endocrine cells. The resemblance of these cells to the human and mouse MPs present during development remains unclear and could have implications for their therapeutic application.

### Bipotent Progenitors

scRNA-Seq analyses supported by embryonic pancreas staining identified new markers of trunk BPs: Anxa2 in mice (Yu et al., 2019) and Dcdc2a in both mice and humans (Scavuzzo et al., 2018). Furthermore, scRNA-Seq revealed that early trunk cells arise between E11.5-12.5 and bifurcate at E14.5 to generate either pro-ductal or pro-EP trunk cell intermediate subpopulations, the cells of this latter population already displaying low levels of *Neurog3* expression (Yu et al., 2019). Simultaneously, the pro-EP E14.5 BPs develop into either EPs or transcriptionally distinct BPs at E16.5 (Scavuzzo et al., 2018). The change in BPs between E14.5 and E16.5 may already reflect a bias toward either an α- or a β-cell fate, as observed in scRNA-Seq experiments (see section “Endocrine Progenitors”). If this is indeed the case, then the change in endocrine cell type must be decided before the onset of Neurog3 expression. This scenario is supported by a another scRNA-Seq study revealing that methylation patterns in the UR2 promoter region of the *Arx* gene, encoding transcription factor determining β- vs. α-cell fate (Dhawan et al., 2011), are established before the expression of its regulator, Neurog3 (Liu et al., 2019). When highly methylated by the Dnmt1 DNA methyltransferase, UR2 adopts an α-cell fate (Liu et al., 2019). Another example supporting for pre-Neurog3 priming is the *Amotl2* gene, which is enriched in pro-β-fate E16.5 compared to pro-α- E14.5 BPs (Scavuzzo et al., 2018), and which is expressed in similar pattern as *Neurog3* (Scavuzzo et al., 2018; Bastidas-Ponce et al., 2019; van Gurp et al., 2019). Amotl2, a Hippo pathway component, inhibits the Notch and canonical Wnt pathways, induces a loss of polarity, and promotes endothelial cell motility (Wang et al., 2011; Zhao et al., 2011; Li et al., 2012; Mojallal et al., 2014). The Notch and canonical Wnt pathways block EP specification (Jensen et al., 2000; Cebola et al., 2015; Rosado-Olivieri et al., 2019; Sharon et al., 2019a). Amotl2 may, therefore, regulate EP specification upstream from Neurog3. *AMOTL2* knockdown in human ESC-derived BP stage cells increases the *GCG* and decreases *INS* expression at the endocrine cell stage. The early differences in Amotl2 expression between pro-α and pro-β BPs may reflect differences in behavior between developing endocrine lineages during delamination, reflecting further spatial organization within islets.

The Wnt, Notch, and Bmp pathways are key regulators of pancreatic endocrine cell development. However, we still do not fully understand signaling pathways regulating endocrine lineage specification. The ERK pathway, for example, was not known to be involved in this process, but scRNA-Seq revealed ERK signaling downregulation when BPs developed into early EPs (Yu et al., 2019). Immunostaining confirmed that pERK was expressed in Neurog3^low^ pro-EP trunk cells but not in Neurog3^high^ EP cells in E14.5 pancreas. Furthermore, transient MAPK/ERK pathway suppression by a small molecule (U0126) in E13.5 pancreatic explants increased the proportion of *Ins1*^+^ and *Gcg*^+^ cells (Yu et al., 2019). Thus, scRNA-Seq has identified the MAPK/ERK pathway as a regulator of mouse endocrine specification. Further studies are required to confirm that ERK signaling enhances human β-cell differentiation *in vitro*.

### Endocrine Progenitors

A combination of lineage tracing and sc-qPCR revealed that as many as 50% of E9.5 pancreatic cells are biased toward the endocrine lineage (Larsen et al., 2017), corresponding to the first wave of endocrine differentiation. By scRNA-Seq, endocrine differentiation from *Ptf1a*^–^ MP-early cells has been observed at E9.5, with the emergence of *Neurog3*^+^ progenitors (“pre−α−first cells”), which further mature from the α−first−early cells at E10.5 to a diverse population of more mature α−first−late cells by E11.5-E13.5 (Yu et al., 2019). The α-first-cells were also identified at E12.5 in a separate study (Bastidas-Ponce et al., 2019). These α−first-cells differ significantly from those of the second wave and have a specific gene signature (Bastidas-Ponce et al., 2019; Yu et al., 2019). The role of the first-wave α-like cells remains unclear, as these cells are not found at later stages in mice. It remains to be determined whether they acquire the characteristics of second-wave α-cells, or undergo apoptosis. A combination of lineage tracing and scRNA-Seq could address some of these questions.

During the second wave at E13.5-E17.5, EPs rapidly transit toward distinct endocrine fates, but multiple subpopulations representing subsequent maturation stages have consistently been identified by scRNA-Seq (Byrnes et al., 2018; Scavuzzo et al., 2018; Bastidas-Ponce et al., 2019; Yu et al., 2019). Importantly, cells with low levels of *Neurog3* expression were detected within the tip, trunk/BPs, and ductal clusters, suggesting that EPs emerge in different domains within the pancreas. The earliest EPs give rise to other EP subpopulations as *Neurog3* expression peaks, followed by late subpopulations with the extinction of *Neurog3* expression. The identification of early, *Neurog3*-Low EPs corroborates lineage-tracing results, showing that these cells as proliferating, long-lived cells (Schonhoff et al., 2004; Bechard et al., 2016). Lineage tracing also indicated despite the priming of *Neurog3*-Low cells for an endocrine fate, they may sometimes acquire a ductal or acinar fate. Thus, *Neurog3*-Low cells must either retain some multipotency or dedifferentiate (Bechard et al., 2016).

On their route to becoming endocrine islet cells, EPs delaminate from the epithelium into the surrounding mesenchyme. It is generally thought that individual delaminated EPs cross the mesenchyme, maturing into endocrine cells, and then aggregate into islets (Larsen and Grapin-Botton, 2017). An alternative model was recently proposed, in which EPs do not fully delaminate from the epithelial cord, instead forming budding peninsula-like structures attached to the cord (Sharon et al., 2019b). In this model, α-committed EPs arise first, lining the peninsula border, followed by β-committed EPs, which bud into the interior of the peninsula. This model is consistent with the final architecture of mouse and small human islets. It has been suggested that large human islets are formed by the coalescence of smaller islets. Peninsula-like buds have also been observed at the EP stage of human ESC differentiation (Sharon et al., 2019b). We have captured a small transient early EP subpopulation of Neurog3^+^ cells from E14.5 mouse pancreas. This N14_2 subpopulation displays a strong enrichment in the expression of epithelial-to-mesenchymal (EMT) genes, including vimentin (*Vim*), possibly reflecting the delamination process (Scavuzzo et al., 2018). These markers are lost in late-EPs and endocrine cells (Scavuzzo et al., 2018; Sharon et al., 2019b). Early EPs undergo a remodeling of adherens and tight junctions. Expression of the epithelial marker E-cadherin therefore transiently decreases but is not entirely abolished (Bakhti et al., 2019; Sharon et al., 2019b). This finding provides conclusive support for the hypothesis that EPs turn on delamination and EMT programs only partially, to enable them to move out of the epithelial cord whilst retaining epithelial characteristics ensuring their attachment to the epithelial cord during islet formation, without individual cells traveling through the mesenchyme.

A *Procr*^+^ pancreatic population was recently identified by scRNA-Seq in 8 week-old adult mice (Wang D. et al., 2020). These *Procr*^+^ cells have a transcriptional profile similar to that of the E14.5 N14_2 subpopulation, with similar EMT characteristics and *Procr* expression (Scavuzzo et al., 2018), and to a subpopulation identified by another E14.5 pancreas scRNA-Seq study (Byrnes et al., 2018). Procr is a surface marker of adult stem cells in other mouse tissues, but not in the pancreas (Wang et al., 2015; Yu et al., 2016; Fares et al., 2017). The Procr+ cells in adult mouse pancreas no longer express *Neurog3* but are pre-endocrine descendants of Neurog3*^+^* progenitors and can give rise to α-, β-, δ-, and PP-cells in adult mice (Wang D. et al., 2020). When cocultured with endothelial cells, Procr+ cells expand *in vitro*, forming 3D clusters and differentiating into functional islet-like organoids. Upon transplantation, these organoids rescue streptozotocin-induced hyperglycemia in mice. The Procr*^+^* cells were the first EP-like adult cells to be cultured long-term *in vitro*. It would be interesting to delineate the ancestry relationship between the fetal Neurog3^+^Procr^+^ and adult Neurog3^–^Procr^+^ pancreatic cells and identify their human counterparts.

The late EPs, in which *Neurog3* expression is decreasing, are marked by high levels of *Fev* expression (Byrnes et al., 2018; Scavuzzo et al., 2018; Bastidas-Ponce et al., 2019; van Gurp et al., 2019; Yu et al., 2019). Fev is a transcription factor activated downstream from Neurog3 in the developing pancreas (Miyatsuka et al., 2009). The *FEV*^+^ population in present in the fetal pancreas, human ESC-derived EPs, and immature endocrine cells (Krentz et al., 2018; Ramond et al., 2018; Veres et al., 2019; Augsornworawat et al., 2020). The *Fev*^+^ late-EPs display an activation of endocrine cell genes including the *Chga*, *Isl1*, *Irx2*, and *Mafb* (Scavuzzo et al., 2018; Yu et al., 2019). Fev^+^ cells may, therefore, represent an intermediate cell state between Neurog3^+^ EPs and more mature endocrine cells.

scRNA-Seq studies have collectively shown that the pancreatic EP subcluster based on developmental stage can be broken down into at least four subpopulations: from early to late EPs. Moreover, these studies have revealed that EPs differ in terms of development potential, displaying a distinct propensity for the generation of α- or β-cells. Several markers have been proposed for the identification of EPs biased toward a specific endocrine cell type. For example, it has been suggested that *Peg10* and *Gng12*, which are expressed in *Fev*^+^ cells at E14.5, could be used to characterize pro-α- and pro-β-EPs, respectively (Byrnes et al., 2018). *Myt1* was recently identified as a marker with enhanced expression in a late EP developmental trajectory branch leading to a β-cell fate (Liu et al., 2019). Lineage-tracing experiments have shown that Neurog3^+^/Myt1^+^ cells are less likely to differentiate into α-cells. However, neither *Neurog3* promoter-driven *Myt1* overexpression nor knockout has a major effect on the β- to α-cell ratio. Interestingly, scRNA-Seq studies of *Neurog3^+^/Myt1^+^* and *Neurog3^+^/Myt1^–^* EPs led to the discovery of a *Dnmt1*-driven increased in the methylation of the UR2 promoter of the *Arx* as a functional characteristic of *Neurog3^+^/Myt1^+^* EPs inhibiting the α-cell fate acquisition (Liu et al., 2019).

EPs are formed continually during the secondary transition, with differences between those formed on different embryonic days (Scavuzzo et al., 2018; Bastidas-Ponce et al., 2019). We have shown that EPs born at E14.5 differ from those born at E16.5 in terms of their transcriptome and epigenome, with the latter revealed by ATAC-Seq (Scavuzzo et al., 2018). This heterogeneity reflects the bias of E14.5 EPs toward α-cells, whereas E16.5 EPs preferentially differentiate into β-cells. This finding is consistent with that of a previous report, in which a conditional, tamoxifen-induced reconstitution of Neurog3 in *Pdx1*^+^ cells in *Neurog3*^–/–^ mice revealed an age-dependent shift in the EP fate from a pro-α to a pro-β bias (Johansson et al., 2007). Other scRNA-Seq have also shown a temporal bias in the formation of α- and β-cells during the second wave of endocrinogenesis (Bastidas-Ponce et al., 2019; Yu et al., 2019). Moreover, E14.5 early EPs are present in the tips, whereas, after plexus-to-duct transition at E16.5, early EPs are found almost exclusively in the trunk domain. It remains unclear whether the tip-derived EPs are biased toward an α-cell fate, whereas trunk-derived EPs are biased toward a β-cell fate, which would be consistent with the timeline of EP and endocrine cell emergence.

Importantly, scRNA-Seq identified genes with similar patterns of expression throughout the generation, maturation and transition of EPs into specific endocrine lineages, revealing new candidate regulators of pancreas development. The use of single-cell technology also made it possible to capture rare early born rare endocrine cell types (i.e., δ-, ε-, and PP-cells) (Byrnes et al., 2018; Krentz et al., 2018; Bastidas-Ponce et al., 2019; Sharon et al., 2019b), and decipher the molecular blueprints of these populations. Moreover, scRNA-Seq confirmed a progressive loss of proliferation capacity and exit from the cell cycle in EPs and their progeny (Krentz et al., 2018; Scavuzzo et al., 2018; Bastidas-Ponce et al., 2019; van Gurp et al., 2019; Yu et al., 2019). The intriguing connection between the neuronal and pancreatic endocrine cell development programs (Arntfield and van der Kooy, 2011) has also been further extended by scRNA-Seq, through the identification of genes with previously known roles in the developing brain but not in the pancreas (Krentz et al., 2018; Bastidas-Ponce et al., 2019).

### Endocrine Cell Maturation

Newborn endocrine cells mature to become fully functional adult cells. Two scRNA-Seq-based studies focused on endocrine cell maturation, analyzing β-cells (both studies) and α-cells (one of these studies) from *Ins1* and *Gcg* reporter strains, respectively, in E17.5—P60 mice (Qiu W.-L. et al., 2017; Zeng et al., 2017). A progressive maturation of β- and α-cells was observed, with the expected loss of known markers of immature endocrine cells and gain of mature endocrine cells markers, together with a decrease in the proportion of proliferative cells. However, these two cell types appear to have different maturation dynamics. β-cells mature steadily over time, with a progressive loss of immature cells, the acquisition of more mature states, and the generation of different postnatal subpopulations. A small population of immature β-cells is retained in adult mice, as revealed by both lineage tracing and scRNA-Seq (Bader et al., 2016; Sachs et al., 2020). By contrast, all postnatal α-cells (P9–P60) cluster together, indistinguishably, separately from E17.5-P0 cells, suggesting that the maturation end-state is acquired earlier in α-cells (Qiu W.-L. et al., 2017). Human endocrine cells display a similar differential pattern of maturation, as shown by a comparison of β- and α-cells from a newborn, toddlers (10 months–4 years old), adolescents, and adults (Avrahami et al., 2020). Newborn β-cells clustered separately from maturing β-cells from toddlers or older individuals. These results for newborns were obtained with cells from a single donor, but they may nevertheless indicate that β-cells do not become functional until after birth. This may be because blood glucose levels are regulated directly by the mother’s cells during gestation. The β-cells of toddlers retain multiple characteristics of immature cells, such as activated TNF and Notch signaling. By contrast, α-cells from the islets of individuals of all ages, from toddlers to adults, were indistinguishable, and displayed a pattern of gene expression common to immature, newborn α-cells, suggesting that maturation involves less pronounced transcriptomic changes in α-cells than in β-cells. This finding may also reflect the greater plasticity and regeneration potential of α-cells *in vivo*. Indeed, α-cells can transdifferentiate into β-cells, in a process driven by FOXO1 inhibition (Chera et al., 2014), or the activation of GABA receptor signaling, which can also be induced by artemisinins, a class of antimalarial drugs (Li et al., 2017). ScRNA-Seq provided evidence of α-cell dedifferentiation in mouse and human islets *ex vivo*, when these islets were treated with FOXO inhibitor, GABA or artemisinin drug, artemether (Marquina-Sanchez et al., 2020). However, FOXO inhibition is not specific to α-cells. It also induces β-cell dedifferentiation, preventing the use of this pathway to restore β-cell mass in situations in which not all β-cells are lost. Detailed comparison of transcriptomes during α- and β-cell maturation might identify cell type-specific maturation factors for the refinement of induced maturation and transdifferentiation.

The markers of immature β-cells include retinol binding protein 4 (*Rbp4*) (Qiu W.-L. et al., 2017; Zeng et al., 2017), which is also highly abundant in the β-cell subpopulations present in the adult human pancreas (Segerstolpe et al., 2016; Mawla and Huising, 2019; Camunas-Soler et al., 2020). A combination of single-cell patch-clamp electrophysiology and scRNA-Seq (Patch-Seq) revealed that the RBP4-rich β-cells in the adult human pancreas have reduced functionality, which manifests in lower levels of Na^+^ channel activity and exocytosis (Camunas-Soler et al., 2020). As revealed by another scRNA-Seq study, *Rbp4* levels are also high in surviving β-cells in a multiple-low-dose model of streptozotocin-induced diabetes (mSTZ) in mice (Sachs et al., 2020). Together with the observed alterations to other pathways in these mSTZ-β-cells, these findings suggest that β-cells dedifferentiate to generate the Rbp4^+^ population in pharmacologically induced diabetes.

Maturing β-cells display a decrease in mTORC1 signaling and pro-proliferative gene expression, and changes to amino-acid metabolism, mitochondrial respiration, and reactive oxidative species response gene expression (Zeng et al., 2017). Similar changes in mTORC1 and mitochondrial metabolism are associated with the poor proliferation capacity of immature β-cells, due to a misfolding mutation in proinsulin, responsible for neonatal diabetes (Balboa et al., 2018).

### The Developing Pancreatic Niche

The embryonic pancreas receives mechanical and chemical signals from surrounding tissues and non-epithelial cells within the pancreas. These signals jointly drive development, regulating the expansion and differentiation of progenitors (reviewed in detail by Cozzitorto and Spagnoli, 2019). For example, mesenchyme was shown to be required for pancreas development as long ago as the 1960s (Golosow and Grobstein, 1962). More recently, the use of sophisticated transgenic mouse models has made it possible to separate out the various developmental roles of the mesenchyme at different timepoints (Landsman et al., 2011) and to identify mesenchyme subpopulations with different pro-endocrine potentials (Cozzitorto et al., 2020). Mesenchymal niche-derived factors are beneficial for hPSC differentiation into pancreatic endocrine cells (Guo et al., 2013; Russ et al., 2016; Mamidi et al., 2018; Cozzitorto et al., 2020). Finally, coculture with mesenchyme promotes the self-renewal of mouse ESC-derived Ngn3^+^ progenitors (Sneddon et al., 2012).

scRNA-Seq studies focused initially on the pancreatic epithelium, but also have shed light on the mesenchymal compartment, revealing its heterogeneity at E12.5-E18.5 (Byrnes et al., 2018; Krentz et al., 2018; Scavuzzo et al., 2018). These studies (Byrnes et al., 2018; Scavuzzo et al., 2018) identified similar mesenchymal populations in the E14.5 pancreas: the largest cluster, consisting of archetypal proliferating mesenchyme cells, a few smaller mesenchymal clusters, myofibroblast-like stellate cells, and proliferating and non-proliferating mesothelial populations. Mesenchymal clusters were differentially enriched in secreted factors. One cluster is enriched in Wnt signaling agonists, chemokines, and ACE2, and another expresses TGFβ, Hippo, and Id pathway components, suggesting different functions for these two clusters. Another mesenchymal cluster in the E12.5-E14.5 pancreas is enriched in the NKX2-5 transcription factor and represents the splenopancreatic mesenchyme surrounding the pancreatic buds showed to be essential for endocrine lineage specification (Hecksher-Sørensen et al., 2004; Cozzitorto et al., 2020). Pseudotime analysis from E12.5-E17.5 suggested that mesothelial cells are the progenitors of mesenchyme subpopulations and stellate cells (Byrnes et al., 2018). This function had already been demonstrated in other organs but had never before been shown for the pancreas. A comparison of E12.5, E14.5, and E17.5 scRNSeq datasets revealed that the mesenchymal subpopulations changed, through differentiation and maturation, or simply disappeared during the course of development (Byrnes et al., 2018), corroborating earlier findings for transgenic models. Between E15.5 and E18.5, the mesenchyme also becomes less heterogeneous (Krentz et al., 2018). It would be interesting to elucidate the developmental role of each mesenchymal subtype, by studying the interactions of each subtype with other pancreatic cell types.

## Insight Into Human Pancreas Development From Pluripotent Models

The sequence of developmental events is highly conserved between species, but there are nevertheless interspecies differences in pancreatogenesis (Nair and Hebrok, 2015). For example, NEUROG3 is transiently and robustly expressed, in two waves, in mice (Gradwohl et al., 2000; Schwitzgebel et al., 2000; Gu et al., 2002), whereas human NEUROG3 expression occurs in single wave (Lyttle et al., 2008; Jennings et al., 2013; Salisbury et al., 2014). The embryonic islet cells of mice are mostly monohormonal, whereas a large proportion of human islet cells are initially polyhormonal (Bocian-Sobkowska et al., 1999; Herrera, 2000; Riedel et al., 2012). Moreover, a comparison of scRNA-Seq of mouse and human β- and α-cells revealed differential expression of multiple genes between these species (Xin et al., 2016). These examples highlight the need to confirm any findings obtained in mice in human models.

Ethical restrictions limit studies on human embryos. As a result, the *in vitro* hPSC differentiation has become a powerful tool to study human pancreatic development. Current hPSC differentiation protocols focus on the generation of functional β-cells, but other endocrine cell types and polyhormonal cells also arise during differentiation (Pagliuca et al., 2014; Rezania et al., 2014; Cogger et al., 2017; Nair et al., 2019; Velazco-Cruz et al., 2019). The *in vitro* differentiation aims to mimic the subsequent embryogenesis stages of β-cell development, by modulating the signaling pathways triggered during this process, including signals arising from non-epithelial pancreatic niche cells not directly incorporated into protocols. The use of suspension (3D) protocols results in a microenvironment more closely resembling *in vivo* embryogenesis than that generated by the planar (2D) cultures initially used. This approach also increases the functionality of hPSC-derived β-cells (Russ et al., 2015; Millman et al., 2016; Velazco-Cruz et al., 2019). *In vitro* differentiation as 3D spheroids is also widely used for the generation of other endoderm derivatives, such as intestine or lung cells (Workman et al., 2016; Múnera et al., 2017; Yamamoto et al., 2017; Miller et al., 2019).

The outcome of hPSC pancreatic differentiation is usually assessed by determining the proportion of cells expressing a limited number of stage-specific markers. As an endpoint assay, the β-cell functionality is evaluated either *in vitro* (in terms of glucose-stimulated insulin secretion) or *in vivo* (the ability to restore glucose homeostasis in diabetic mice). However, scRNA-Seq on the developing pancreas have revealed progenitor heterogeneity, including early acquired biases toward progeny lineages and continuous changes in cell state. The use of single-cell omics approaches to study subsequent steps of human *in vitro* pancreatic development more closely would improve the control of pancreas engineering, paving the way for clinical applications.

To this end, scRNA-Seq has been used to specify populations at different stages during 2D and 3D β-cell differentiation (Krentz et al., 2018; Sharon et al., 2019a; Veres et al., 2019). The 3D protocols used in these studies included six stages, with PDX1^+^ pancreatic progenitors (PP1) forming at the end of stage 3 (8 days of differentiation, efficiency > 90%), PDX1^+^/NKX6-1^+^ progenitors (PP2) at the end of stage 4 (5 more days, ∼60% efficiency), and immature NKX6-1^+^/C-PEP^+^ endocrine cells (EN) forming at the end of stage 5 (7 more days), followed by reaggregation to promote maturation into functional β-cells (SC-β) after seven or more additional days. At the PP1 stage, the PDX1^+^ population was rather homogeneous, with the exception of a discrete PDX1^+^ subpopulation undergoing mitosis, corresponding to high differentiation efficiency at this stage (Veres et al., 2019). At the PP2 stage, PDX1^+^/NKX6-1^+^ progenitors constituted the largest population, including a proliferating subpopulation, followed by EPs (NEUROG3^+^ and FEV^+^ISL^–^ populations), first α-like cells, and a rare SST^+^/HHEX^+^ δ-like population. The presence of EPs and their derivatives at this early stage indicates a precocious induction, before the NKX6-1 expression (Russ et al., 2016; Sharon et al., 2019a), and the initiation of an α-like differentiation, probably leading to bihormonal GCG^+^/INS^+^ cells. NEUROG3^+^NKX6-1^–^ and NKX6-1^–^ endocrine cells were also detected early in 2D pancreatic differentiation by sc-qPCR and scRNA-Seq (Petersen et al., 2017; Krentz et al., 2018). The endpoint endocrine cells were mostly polyhormonal and immature relative to adult human islet cells. It would be interesting to determine whether these cells correspond to the first wave of α-cells from mouse early MPs, which has yet to be clearly demonstrated in human pancreas development, or to the second wave of endocrine differentiation, in which α-cells precede β-cells.

scRNA-Seq revealed that PP2 stage progenitors express BMP pathway genes. BMP blocks the precocious induction of *NEUROG3*, whereas BMP inhibition promotes this induction (Sharon et al., 2019a). BMP inhibitors were widely included in the early 2D and 3D differentiation protocols. scRNA-Seq also revealed an enrichment in canonical Wnt pathway components in PPs relative to endocrine-committed cells. By contrast, endocrine cell clusters display an enrichment in APC, which inhibits Wnt, suggesting that Wnt signaling may suppress endocrine induction (Sharon et al., 2019a). Indeed, the preservation of Wnt activity in *Neurog3*^+^ cells, via conditional Cre-mediated APC KO, blocks their differentiation into endocrine cells in mice (Sharon et al., 2019a). Conversely, the Wnt inhibition at the EN stage substantially increases the efficiency of C-PEP^+^/NKX6.1^+^ cell derivation (Sharon et al., 2019a).

Unexpectedly, scRNA-Seq identified a novel endocrine population arising from *NEUROG3*^+^ progenitors during the EN stage, along with immature β-, α- and rare δ-like cells (Veres et al., 2019). The cells of this SC-EC population, resemble enterochromaffin cells, a serotonin-producing chemosensor gut cell type (Grün et al., 2015; Haber et al., 2017). SC-ECs have a frequency similar to that of SC-β cells (Veres et al., 2019) and can be identified by scRNA-Seq in 2D differentiation (Krentz et al., 2018; Augsornworawat et al., 2020). SC-ECs appear to be closely related to SC-β cells, which can also produce serotonin *in vivo* (Almaça et al., 2016). They also have a similar developmental program to common *NEUROG3*^+^ progenitors, suggesting possible misdifferentiation *in vitro*, as these cells do not seem to arise in the pancreas *in vivo* (Veres et al., 2019; Augsornworawat et al., 2020). Moreover, enterochromaffin marker genes were induced in a β-cell dedifferentiation model (Lu et al., 2018; Veres et al., 2019), again pointing to a close relationship between the two cell types. SC-ECs express *CHGA*, *NKX6-1*, and low levels of *INS*, but they do not express *GCG* (Veres et al., 2019), suggesting possible misalignment with β-cells in immunostaining-based assays. The depletion of SC-ECs with the CD49a surface marker, identified by scRNA-Seq as characteristic of SC-β population, results in an enrichment in SC-β cells and promotes their maturation (Veres et al., 2019). Other groups have similarly observed that reaggregation (based on the INS-GFP reporter) or simple cluster resizing promotes the *in vitro* β-cell maturation (Nair et al., 2019; Velazco-Cruz et al., 2019). As enrichment for β-like cells based on a surface marker or reporters cannot be applied to clinical-grade preparations, it would be desirable to block SC-EC during differentiation. Single-cell transcriptomics has provided insight into the bifurcation event, during which common NEUROG3^+^ progenitors split into two groups destined to become either SC-EC or SC-β cells; over 300 genes displaying enriched expression in one particular branch were identified (Veres et al., 2019), providing a possible starting point for protocol refinement.

Interestingly, scRNA-Seq also revealed that significant proliferative non-endocrine populations appear first at the EP stage and then at the endocrine maturation stage. These non-endocrine cells develop into ductal, acinar and mesenchymal cell-like populations (Veres et al., 2019), implying that some of the PDX1^+^/NKX6-1^+^ cells at the PP2 stage retain or regain multipotency. This hypothesis is supported by lineage-tracing in mice showing that early progenitors with Neurog3 low expression retain a small degree of multipotency (Bechard et al., 2016). The 3D sphere dissociation and reaggregation at EP stage purifies endocrine cells from non-endocrine populations (Veres et al., 2019). However, protocol refinement to block non-endocrine commitment would probably increase the yield of functional β-cells.

Mechanosignaling from the mesenchyme is critical for PP fates (Mamidi et al., 2018). During development, the mesenchymal extracellular matrix triggers F-actin depolymerization, leading to the recruitment of YAP1 to the cytoplasm, impeding its nuclear function in the blocking of *NEUROG3* expression together with Notch. scRNA-Seq was used to investigate the involvement of cytoskeleton remodeling in EP induction (Hogrebe et al., 2020). In the experimental conditions used, almost all the adherent multipotent PDX1^+^ progenitors developed into PDX1^+^/NKX6-1 BPs within 5 days. However, F-actin depolymerization by latrunculin A greatly decreases the proportion of PP2 (about 20% of all cells) and stimulates precocious EP differentiation (about 50%) without the NKX6-1 induction, and leads to the an undefined endodermal fate (about 26%), probably non-pancreatic. Conversely, reinforcement of the F-actin skeleton by nocodazole treatment shifted differentiation toward exocrine-like progenitors (about 66% of all cells). The application of latrunculin A during the first day of a seven-consecutive day stage in which BPs differentiate into EPs and immature endocrine cells leads to the efficient generation of functional β-cells from various cell lines, mostly in 2D protocols. Lantrunculin-induced cytoskeleton alterations also affect differentiation into other primitive gut descendants, such as intestine and liver cells, suggesting that the possible mechanosignaling mechanisms common to the development of other endodermal organs (Hogrebe et al., 2020). The SC-β cells generated by planar protocols are functional *in vitro* and rescue STZ-induced diabetes in mice similarly to human islets. However, these SC-β cells retain multiple transcriptomic and functional features of juvenile, immature β-cells, and are thus different from human adult islets (Augsornworawat et al., 2020; Hogrebe et al., 2020). *In vivo*, SC-β cells further mature toward a more human islet-like state, as revealed by scRNA-Seq 6 months after transplantation (Augsornworawat et al., 2020). A closer look at the pathways active in grafted SC-β, might reveal novel candidate regulators of maturation.

The advent of CRISPR technology has increased the accessibility of genetically engineered hPSCs, allowing the manipulation of known or putative regulators of development for their function assessment in the human context. Again, scRNA-Seq may facilitate characterization of the mutation consequences. Russell et al. recently assessed the developmental role of MAFB (Russell et al., 2020), a transcription factor that binds to crucial β-cell genes, including *INS* and *PDX1* (Nishimura et al., 2006; Vanhoose et al., 2008). This is particularly interesting given the differential expression of *MAFB* between species. It is expressed in human adult α-, β- and δ-cells (Fang et al., 2019; Avrahami et al., 2020), whereas it is expressed solely in α-cells in adult mice, its expression in β-cells being lost upon maturation of these cells (Qiu W.-L. et al., 2017; Zeng et al., 2017). MAFB murine KO results in a quantitative and functional α-cell deficiency, whereas β-cell development is only delayed (Katoh et al., 2018). As demonstrated by scRNA-Seq, MAFB KO does not affect hPSC-to-EP differentiation; instead, it blocks α- and β-cell specification, leading to the induction of rare endocrine lineages, such as δ-cells, PP-cells, gastrin^+^ and peptide YY^+^ cells (Russell et al., 2020). No link has been established between MAFB mutations and diabetes, but many other genes have been shown to cause monogenic forms of diabetes. Thus, hPSC lines with KOs of these genes, and patient-derived iPSCs are potentially invaluable models for research into pancreas development and diabetes pathogenesis.

β-cells initially proliferate, exiting the cell cycle once they have matured (Kulkarni et al., 2012). However, a low level of proliferation is maintained in adulthood, to allow for β-cell mass maintenance (Bader et al., 2016). The identification of rare adult β-cells capable of proliferating, and the characterization of triggers and signaling pathways via which β-cells re-enter the cell cycle are of considerable interest, as a way of replenishing these cells in diabetes patients *in vivo*, and of increasing the yield of *in vitro*-derived β-cells. The YAP pathway was shown to induce SC-β cell proliferation (Rosado-Olivieri et al., 2019). Rosado-Olivieri made use of this discovery to force SC-β-cells to re-enter the cell cycle, and then used scRNA-Seq to identify the enriched pathways, as putative proliferation drivers (Rosado-Olivieri et al., 2020). One of the identified drivers was the leukemia inhibitory factor (LIF) pathway, acting through JAK/STAT and the CEBPD transcription factor. LIF induces the proliferation of mouse β-cells *in vivo*, human islets grafted into murine kidneys, and SC-β-cells. Interestingly, LIF receptor (*LIFR*)-positive β-cells constitute a small (less than 20%) subpopulation of cells with a distinct transcriptomic profile among SC-β cells or adult human islets (Rosado-Olivieri et al., 2020).

In addition to β-cell degeneration, α-cell dysfunction may also underlie diabetes progression (Gromada et al., 2018; Yosten, 2018). In the T1D treatment, α-cells are essential for the tight control of islet hormone secretion, and for the regulation of glucose levels, but most studies to date have focused on the generation of β-cells. Early NEUROG3 induction in PDX1^+^/NKX6-1^–^ progenitors leads exclusively to the generation of α-like bihormonal cells. BMP inhibition has therefore been used to enhance this induction, resulting in the efficient generation of functional α-like cells, known as SC-α cells (Peterson et al., 2020). The bihormonal α-like cells (pre-α cells) express both GCG and INS, but pro-insulin is not processed to generate mature insulin in these cells (Peterson et al., 2020). Bihormonal α-like cells are transiently present during human pancreatic development and in diseases, such as diabetes (Riedel et al., 2012; Md Moin et al., 2016). The pre-α cells eventually mature into monohormonal GCG^+^ cells. *In vitro*, the functional maturation of these cells is efficiently promoted PKC activator treatment during stage 6. By contrast, the pre-α-cells maturation into monohormonal SC-α cells was rare in prior protocols for β-cell maturation. A comparison of the transcriptomes of individual pre-α-cells and mature SC-α cells revealed a subtle maturation process, with the silencing of stress-related and insulin secretion pathways and the induction of glucagon release [(Peterson et al., 2020). Studies based on pre-α-cell transplantation (Augsornworawat et al., 2020) have suggested that SC-α cells undergo further maturation in mice. Such a transplantation model could be used to identify regulators of α-cell maturation, leading to further refinement of the *in vitro* derivation protocol.

## Diabetes

scRNA-seq provides insight into the diabetes-induced dysfunction of each islet cell type and subtype, making it possible to identify pathway dysregulations undetectable with bulk RNA-seq. Main studies that used scRNA-Seq to identify pathways involved in diabetes or obesity are summarized in Supplementary Table 2. In the initial studies, T2D-related transcriptional alterations were found not only in β-cells, but also in other islet and non-endocrine cells (Segerstolpe et al., 2016; Xin et al., 2016; Lawlor et al., 2017). In addition, there is growing evidence to suggest that adult endocrine cell populations are not homogeneous (Supplementary Table 2) and that the ratios of the various subpopulations change in pathogenic situations. Adult β-cells differ in terms of their phenotypic, proliferative, and functional characteristics, and these cells may have different sensitivities to glucose. Multiple molecular markers have been proposed for the characterization of β-cell heterogeneity (Dominguez-Gutierrez et al., 2019). For example, a small population of β-cells with pacemaker properties, called hub cells, has been identified (Johnston et al., 2016). These cells with immature characteristics are indispensable for the islet-wide coordinated response to glucose and are specifically targeted by diabetic stress. scRNA-Seq could, therefore, potentially reveal alterations in specific subpopulations.

A comparison of the transcripts dysregulated in T2D from multiple studies revealed a minimal overlap of genes, possibly due to the limited numbers of donors and of sequenced cells (Wang and Kaestner, 2019). As only a fraction of the cells may be affected by disease, and changes in the expression of individual genes may be subtle, transcriptional dysregulation may be masked by natural variation between individuals. Fang et al. therefore developed the RePACT (regressing principal components for the assembly of continuous trajectory) strategy, in which changes in cellular heterogeneity in a context of obesity or T2D were treated as a development-like pseudotime trajectory (Fang et al., 2019). The combination of this approach with high-throughput scRNA-Seq resulted in the identification of discrete affected β-cell subpopulations, the comparison of which increased statistical power and made it possible to detect changes in gene expression common to obesity and T2D and changes specific to each of these conditions. The common alterations included an upregulation of hypoxia-related genes and a downregulation of aerobic respiration-related genes. *INS* was also one of the genes deregulated, displaying upregulation in obesity but downregulation in T2D, confirming previous scRNA-Seq findings (Segerstolpe et al., 2016). Other genes with inverse patterns of expression in obesity and T2D included two ferritin genes, *FTL* and *FTH1*, encoding proteins involved in intracellular iron metabolism (Fang et al., 2019). This finding is consistent with the observation that obese patients have low serum iron levels, whereas high serum iron levels are a risk factor for T2D (Simcox and McClain, 2013). In addition, a combination of the RePACT approach and a CRISPR screen identified known and unknown insulin regulators in T2D and obesity trajectories, including Mau2-Nipbl cohesin loading complex, a new *INS* gene transcription regulator, and the NuA4/Tip60 HAT complex, a new insulin secretion regulator, with possible roles in diabetes development (Fang et al., 2019).

Another insight into compensatory mechanisms related to β-cell physiology in T2D was provided by a powerful multimodal approach, in which single-cell transcriptomes for endocrine cells were coupled with exocytosis measurements, for the estimation of glucose-dependent insulin secretion with Patch-Seq technology (Camunas-Soler et al., 2020). Multiple genes with expression patterns positively or negatively correlated with exocytosis in healthy cells were inversely correlated with exocytosis in individuals with T2D. These results suggest that damaged islet cells display changes in functionality-related transcript levels with increases in blood glucose levels, but that their response is weak. One of the mechanisms thought to underlie this phenomenon is an increase in inflammation due to the insufficient degradation of ETV1 and STAT3 by COP1 ubiquitin ligase (Suriben et al., 2015; Nordmann et al., 2017). Indeed, ETV1 knockdown increases exocytosis β-cells from T2D patients, but not in healthy β-cells (Camunas-Soler et al., 2020). It has been suggested that hyperglycemia-induced inflammation triggers endocrine cell dedifferentiation in T2D and T1D (Talchai et al., 2012; Cinti et al., 2016; Nordmann et al., 2017; Bensellam et al., 2018; Seiron et al., 2019). In support of this dedifferentiation model, single-cell studies performed by the group of Kaestner have shown that the gene expression patterns of α- and β-cells from T2D individuals are similar to that of juvenile, immature cells, suggesting that damage to endocrine cells triggers dedifferentiation (Wang et al., 2016; Avrahami et al., 2020). Furthermore, a comparison of the transcriptome of immature or mature β-cells from healthy, developing mice with that of β-cells surviving mSTZ, revealed a reversion to an embryonic immature β-cell-like state (Sachs et al., 2020). Years after the onset of T1D, only a few β-cells prevail in most patients (Keenan et al., 2010; Oram et al., 2015), suggesting that β-cell redifferentiation is a promising strategy for the treatment of T1D and T2D.

Dedifferentiation probably involves epigenetic mechanisms. Based on CHIP-Seq and scRNA-Seq in mice, Lu et al. recently identified a loss of polycomb repressor complex (PRC2) function as the underlying cause of adult β-cell identity loss (Lu et al., 2018). They confirmed the PRC2 dysfunction in T2D islets. Mechanistically, the dysregulation of PRC2 in β-cells triggers epigenetic remodeling at specific loci in the β-cell genome in the context of a high-fat diet, in mice with a loss of PRC2 function, resulting in an increase in transcriptional entropy, with the ectopic expression of bivalent loci and an explicit loss of the acetylation of β-cell-specific transcription factors (Lu et al., 2018). The re-activation of bivalent loci characteristic of immature β-cells in human newborns has also been observed in T2D β-cells (Avrahami et al., 2020).

Diabetes induced by stress-activated β-cell dedifferentiation may be reinforced by the inhibition of FOXO1 signaling (Talchai et al., 2012). scRNA-Seq in *ex vivo* mouse and human islets treated with FOXO1 inhibitor demonstrated β-cell dedifferentiation revealed as similar dedifferentiation of α-cells (Marquina-Sanchez et al., 2020). In juvenile mice depleted of β-cells, FOXO1 inhibition also results in δ-cell dedifferentiation to such an extent that these cells can undergo endocrine lineage specification (Chera et al., 2014). A recent study by Sachs and colleagues showed that combined treatment with PEGylated-insulin and GLP-1-estrogen conjugate reverses STZ-induced diabetes, mainly by inducing the proliferation and redifferentiation of dedifferentiated β-cells (Sachs et al., 2020). The FOXO1 and MAPK pathways were implicated in this process.

iPSCs derived from diabetic patients are a powerful tool for studies of pathogenesis and efforts to develop effective treatments. In T2D or monogenic diabetes, patient iPSC-derived β-cells or islets can be used for autologous transplantation (after genetic correction in the case of monogenic diabetes). For studies on the mechanisms underlying disease, candidate gene mutations can be introduced into healthy PSCs. Progress in diabetes research through studies using human PSC models has been described in detail elsewhere (Balboa et al., 2018). More recently, scRNA-Seq has been used to determine the precise effects on β-cell differentiation of mutations in patient-derived iPSCs (Balboa et al., 2018; Maxwell et al., 2020). Notably, iPSCs carrying mutations have been compared to genetically corrected isogenic lines, a method of decreasing intra-iPSC line variability. In a first study, iPSCs with a heterozygous *INS* C96Y mutation resulting in proinsulin misfolding and neonatal diabetes differentiated into β-like cells less efficiently than their counterparts with a corrected mutation. scRNA-Seq revealed high levels of endoplasmic reticulum (ER) stress, low levels of mTORC1 signaling, and mitochondrial alterations, which together resulted in early proliferation defects in cells with misfolded C96Y proinsulin, where were validated following the transplantation of these cells into mice (Balboa et al., 2018). The restoration of mTORC1 signaling in a mouse model of insulin misfolding, the Akita mouse, rescued neonatal β-cell proliferation defects and aggravated the diabetic phenotype (Riahi et al., 2018). These studies together identify mTORC1 signaling as a potential treatment target in neonatal diabetes, although any treatment would need to be applied very early in life to be effective. In another study, the CRISPR-Cas9 system was used to correct a diabetes-causing pathogenic variant of the Wolfram syndrome 1 (WFS1) gene in iPSCs derived from a patient with Wolfram syndrome (WS) (Maxwell et al., 2020). This correction improved the differentiation and maturation of SC-β cells relative to uncorrected isogenic cells, including their ability to secrete insulin in a dynamic manner following stimulation with glucose *in vitro*, and it reversed diabetes following the transplantation of corrected cells into a diabetic mouse. A comparison of scRNA-Seq transcriptomes from corrected and uncorrected iPSCs at the mature endocrine cell stage revealed substantial misdifferentiation, with an expansion of the non-pancreatic and acinar lineages at the expense of SC-β, α-, and δ-cells, and most of the remaining endocrine cells being enterochromaffin cells. Patient SC-β cells experienced higher levels of ER and mitochondrial stress than corrected cells, and displayed higher levels of expression for apoptosis markers, as generally observed in cases of WFS1 gene deficiency (Fonseca et al., 2005, 2010; Yamada et al., 2006) and consistent with a previous Wolfram Syndrome iPSC study (Shang et al., 2014). Collectively, scRNA-Seq has greatly extended our knowledge of the mechanisms involved in endocrine cell dedifferentiation in diabetes, paving the way for β-cell mass restoration.

## Non-Diabetic Diseases of Pancreas

In this review we mainly focus on endocrine pancreas, however, diabetes is often strictly linked to other prevalent pancreatic conditions, like cancer and pancreatitis. These conditions share a common feature—the long-term pancreatic inflammation and, together with obesity, significantly increase risk to develop each other (Paternoster and Falasca, 2020). Pancreatic ductal adenocarcinoma (PDAC) is one of the leading death-causing cancer types worldwide, yet early malignancy markers allowing detection and treatment at pre-metastatic stage and prior to drug resistance acquisition are missing (Storz and Crawford, 2020). PDAC develops from benign lesions through a complex interplay between transformed exocrine cells and microenvironment, as it has been thoroughly reviewed elsewhere (Bulle and Lim, 2020; Storz and Crawford, 2020). The two essential components for PDAC development are: (1) acquisition of an oncogenic mutation by acinar or ductal cells, with KRAS oncogene counting for 90% of cases; (2) inflammation within microenvironment which drives early lesion development. Multiple scRNA-Seq studies in PDAC mouse models and human tumor samples shed light on tumor cell identity and heterogeneity, as well as tumor microenvironment (TME), including role of cancer associated fibroblasts (CAFs) and immune landscape, and cross-talk between tumor and TME (Ting et al., 2014; Bernard et al., 2019; Elyada et al., 2019; Hosein et al., 2019; Ligorio et al., 2019; Peng et al., 2019; Dominguez et al., 2020; Hwang et al., 2020; Lee et al., 2020; Schlesinger et al., 2020; Steele et al., 2020; Zhou et al., 2021). These studies showed that intra-tumor heterogeneity identified by scRNA-Seq is reliable as a prognostic marker and can be used for personalized treatment choice. Importantly, scRNA-Seq on samples collected by fine-needle low-input biopsies is sufficient as diagnostic tool (Lee et al., 2020; Steele et al., 2020). Moreover, these studies revealed acquisition of immunosuppressive TME during neoplastic progression, which allow to further focus on mechanisms beyond this phenomenon.

PDAC can develop when control over a physiological regeneration of exocrine pancreas fails (Storz, 2017). The regeneration is driven by a reversible process of acinar cells dedifferentiation into duct-like exocrine progenitors (acinar-to-ductal metaplasia, ADM), and the transitional, plastic populations were observed in adult human pancreata and PDAC samples by scRNA-Seq (Qadir et al., 2020; Zhou et al., 2021) and single-nucleus RNA-Seq (sNuc-Seq) (Tosti et al., 2021). The irreversible dedifferentiation and unrestrained proliferation of these progenitors leads to pancreatic intraepithelial neoplastic lesions (PanIN) and eventually PDAC (Storz, 2017). Regenerating gene protein (REG) family-enriched acinar cells were identified as promoting ADM and PanIN growth (Gironella et al., 2013; Liu et al., 2015). A small proportion of REG+ population was identified by scRNA-Seq also in non-disease pancreata (Muraro et al., 2016; Tosti et al., 2021), and enriched in low-grade lesions (Bernard et al., 2019) and PDAC (Schlesinger et al., 2020). A recent sNuc-Seq study identified REG+ cells as the only exocrine cluster in a pancreatitis sample, whereas they were minority in healthy pancreas and not present in neonatal pancreas (Tosti et al., 2021). *In situ* sequencing revealed that these cells reside near macrophages, possibly modulating immune response (Tosti et al., 2021). Moreover, research on PDAC, pre-metastatic PanIN and intraductal papillary-mucinous neoplasms, identified transition of KRT19+ ductal populations along tumor development, where MUC5AC-rich clusters are present in benign lesions, and MUC5B+ metaplastic populations marks pancreatitis and PDAC (Bernard et al., 2019; Peng et al., 2019; Schlesinger et al., 2020; Tosti et al., 2021).

Several recent scRNA-Seq studies focused on role of TME components, for example CAFs, in PDAC development. CAFs are actively involved in tumor growth, metastasis and drug resistance in various types of cancers (Bulle and Lim, 2020; Hosein et al., 2020). Myofibroblastic myCAFs and inflammatory iCAFs were recently described as two subtypes present in PDAC and originating from activated pancreatic stellate cells (Öhlund et al., 2017). These cells were robustly identified at different stages of lesion development by multiple scRNA-Seq studies (Bernard et al., 2019; Elyada et al., 2019; Hosein et al., 2019; Ligorio et al., 2019; Peng et al., 2019; Dominguez et al., 2020). Moreover, a novel subpopulation of antigen presenting CAFs (apCAFs) was discovered by scRNA-Seq study (Elyada et al., 2019) and further confirmed by others (Hosein et al., 2019; Dominguez et al., 2020; Lee et al., 2020; Zhou et al., 2021). The scRNA-Seq data led to further insights into CAFs roles in tumor development, e.g., changes the subtypes proportions during lesion progression and after drug treatment (Bernard et al., 2019; Dominguez et al., 2020; Zhou et al., 2021), immunosuppressive roles of iCAFs and apCAFs (Bernard et al., 2019; Elyada et al., 2019), identification of CAF-specific prognostic markers like LRRC15^+^ (Dominguez et al., 2020), identification of TGFβ as CAF-released signal inducing metastatic and proliferative PDAC phenotype run by MAPK and STAT3 pathways (Ligorio et al., 2019), and suggested CAFs plasticity as a potential therapeutic target (Feldmann et al., 2021). These advanced scRNA-seq studies together provided valuable input into the molecular mechanism of PDAC, and provided novel candidate targets for treatment.

## Discussion: Concluding Remarks and Future Perspectives

Many studies have shown that scRNA-Seq technology has the power to confirm and extend the fundamental findings obtained with traditional research approaches. Moreover, the datasets generated by different research groups, with platforms using advanced and less advanced technologies, or different patterns of cell-type enrichment, have generally been similar, after variability in sensitivity, technical noise, and data processing methods has been taken into account. Multiple datasets for the developing pancreas have made it possible to identify equivalent, previously unknown pancreatic subpopulations. Furthermore, the use of novel bioinformatic tools to re-analyze existing datasets and integrate them into a single model may reveal details that had previously been missed. For example, the integration of adult pancreas datasets resolved clusters of sporadic populations, such as ε, Schwann, mast and macrophage cells, that had remained undetected in the individual datasets (Stuart et al., 2019).

**FIGURE 2:**
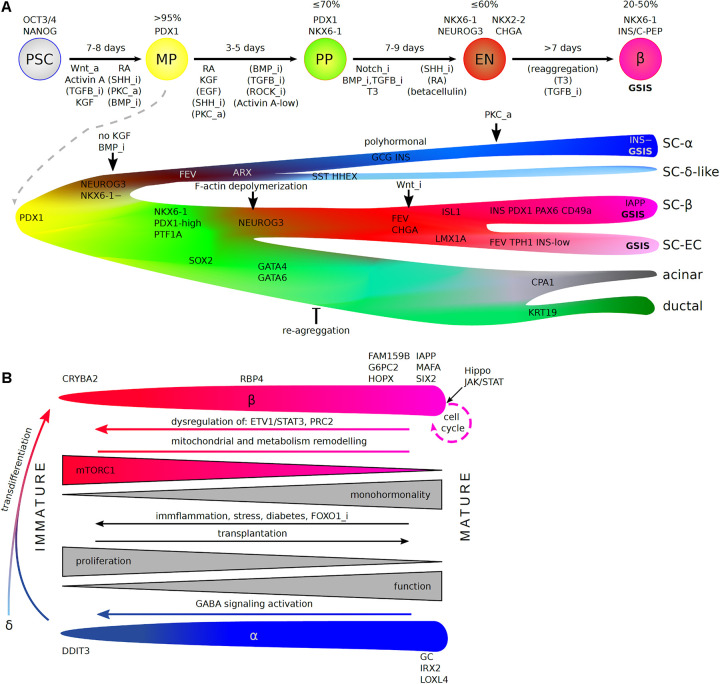
scRNA-Seq insight into *in vitro* hPSC differentiation toward human pancreatic β-cells **(A)** and endocrine cell maturation **(B)**. **(A)** *(Top)* Stages of differentiation protocols (arrows), which recapitulate consecutive pancreas development steps *in vivo* (circles). Denoted are: a length of each stage (above arrows, in days), genes commonly used to estimate differentiation efficiency (% + for stage markers out of all cells), and pathways that are inhibited (“_i”) or activated (“_a”) during each stage (below arrows). Pathways without brackets are essential for the process and applied in all commonly used protocols, while brackets indicate pathways regulated in a fraction of protocols. PSC—pluripotent stem cells, MP—multipotent progenitors, BP—bipotent progenitors, EN—endocrine lineage (endocrine progenitors and immature endocrine cells), β—β-cells, GSIS—glucose stimulated insulin secretion. *(Bottom)* A detailed view on the differentiation based on scRNA-Seq reveals the origin of non-β-cell specific populations that deteriorate differentiation efficiency and points to branching at which the protocols could be refined. Factors identified in scRNA-Seq studies that improve specific lineage choices are denoted. SC—stem cell-derived, EC—enterochromaffin cells. **(B)** Maturation of β- and α- cells including molecular changes and marker genes along the process. Dedifferentiation (reverse arrows), transdifferentation, and re-entering cell cycle are possible as physiological compensatory mechanisms, in pathology and when artificially forced by identified factors, with potential use in medicine.

The ultimate aim of endocrine pancreatic research is the development of better treatments or cures for diabetes. In this context, an increasing number of scRNA-Seq studies are identifying candidate regulatory genes and pathways beyond the development and pathogenesis of diabetes, for which further validation is required. The curated data for the developing pancreas will make it possible to refine *in vitro* and *in vivo* methods for manipulating pancreatic endocrine cell identity and expansion. For example, ligand-receptor connectome analyses can be performed on existing time-series datasets including pancreatic niche components, to identify time-dependent, specific niche-derived signals influencing cell-fate decisions (Byrnes et al., 2018; Scavuzzo et al., 2018). The power of such systems biology approaches was recently demonstrated in an elegant study by Han et al., in which the authors used scRNA-Seq to reveal complex interactions, including some involving signaling pathways, between the gut tube endoderm and mesoderm at E8.5-E9.5 (Han et al., 2020). In the human context, scRNA-Seq has highlighted a number of obstacles to the robust generation of functional human β-cells, including: (1) the precocious differentiation of NKX6-1^–^ pancreatic progenitors, (2) the incomplete commitment of PDX1^+^NKX6-1^+^ cells to an endocrine fate, and (3) misdifferentiation into enterochromaffin-like cells (Figure 2A). An analysis of the bifurcation events underlying these unwanted fates, even with existing datasets, might yield sufficient candidate regulators for the improvement of *in vitro* differentiation. For example, the discovery of precocious α-cell specification led to the development of a protocol for functional SC-α cell generation (Peterson et al., 2020). The combination of α- and β-cells derived from cell-specific protocols into single islets could also improve *in vivo* maturation and function.

Single-cell research on tissues from diabetic patients, and in human and mouse genetic models, has identified multiple pathways involved in endocrine failure in the pancreas. In particular, such studies have highlighted the occurrence of stress-related mature cell identity loss in surviving endocrine cells, resulting in dysfunctional β-cells unable to respond correctly to glucose stimulation (Figure 2B). The use of single-cell omics approaches has shown that treatment with PEGylated-insulin and GLP-1-estrogen conjugate stimulates β-cell redifferentiation and restores β-cell mass by proliferation, rendering this approach potentially very promising for the treatment of T1D and T2D. Immature endocrine cells have been observed in post-mortem tissues from patients, diabetic mouse models and following specific dedifferentiation-inducing treatments in cell and mouse models. But what if, in some cases of diabetes, the observed immature endocrine cells result from defective pancreas development rather than dedifferentiation? Studies of iPSCs from neonatal diabetes and WS1 patients pointed to defective differentiation *in vitro* and multiple MODY-causing genes. Indeed, defects of pancreatic development have recently been implicated in the pathogenesis of T2D (Perng et al., 2019; Stein et al., 2019) and T1D (Phillips et al., 2017). It may appear counterintuitive to attribute a developmental character to diseases of adulthood, but late-onset neurodegenerative diseases provide an example supporting the consideration of similar hypotheses for diabetes. There are multiple lines of evidence to suggest that neurodegenerative diseases of adults, such as Huntington’s disease (Wiatr et al., 2017), Parkinson’s disease (Schwamborn, 2018) and Alzheimer’s disease (Arendt et al., 2017), are not solely neurodegenerative, but also have a neurodevelopmental component. In Huntington’s disease, abnormal neural development is observed during the hPSC differentiation (Conforti et al., 2018; Smith-Geater et al., 2020) and in affected fetuses and children, years before the disease onset (van der Plas et al., 2019; Barnat et al., 2020). These findings suggest that the disease persists in a latent phase for years, during which compensatory mechanisms within the organ prevent the onset of disease. Similarly, for diabetes, immature or misdifferentiated islets might be more prone to environmental stress induced by an unhealthy lifestyle and impaired glucose homeostasis. Indeed, the pacemaker-like hub β-cells, are preferentially targeted by diabetic stress, leading to functional failure of the whole islet (Johnston et al., 2016). Limited self-renewal of β-cells might lead to a temporary replenishment of the β-cell pool, but the number of these cells gradually declines, eventually leading to diabetes, with an onset earlier or later in life, depending on the strength of the developmental deficits and environmental stress. Research in presymptomatic patients is almost impossible, but these subtle changes in endocrine cell maturity could be evaluated with single-cell approaches in mouse or human PSC-derived pancreatic models. If such developmental defects are found to underlie diabetes, it may be possible to develop a pre-onset treatment for patients with a known genetic or environmental predisposition to diabetes.

The ongoing development of scRNA-Seq technologies is improving sequencing yields and depths, facilitating more accurate biological inference (Ding et al., 2020). It is particularly difficult to study individual cells in the pancreas, due to the high hydrolytic enzyme content of the exocrine cells. The almost exclusive removal of the exocrine pancreas hinders scRNA-Seq research on acinar and ductal cells in the adult pancreas. Methods for overcoming these limitations are emerging, such as snap-freezing of the dissected pancreas followed by single-nucleus RNA-Seq (Tosti et al., 2021). This is also important in the context of a limited availability of biological material, from T1D patients for example, as the use of frozen tissues extends this availability. Also, pancreatic differentiation of patient-specific iPSCs offers alternative platform to study transcriptomes of T1D specific single islet cells. Moreover, single-cell omics techniques other than scRNA-Seq have been developed and their application in combination with scRNA-Seq adds additional layers of information to the existing data (Efremova and Teichmann, 2020), as for Patch-Seq, for example (Camunas-Soler et al., 2020). Furthermore, scRNA-Seq can be combined with scDNA barcoding, using CRISPR for lineage tracing (Raj et al., 2018; Spanjaard et al., 2018), or with sc-epigenetics (Ai et al., 2019; Chen et al., 2019; Li et al., 2019; Rooijers et al., 2019; Zhu et al., 2019). Single-cell proteomics is also being extensively developed (Goltsev et al., 2018; Gut et al., 2018; Saka et al., 2019; Labib et al., 2020). *In situ* single-cell sequencing methods can identify spatial connections between cell types of interest that may be particularly useful for further inferences concerning the structural development of the pancreas or niche and pancreas crosstalk. The *in situ* sequencing of cytoplasmic transcripts can be performed on the same tissue preparations as used for single-nucleus RNA-Seq (Tosti et al., 2021). In summary, pancreas research has already greatly benefited from scRNA-Seq (Table 1). The refined analysis of already curated datasets and the use of emerging technologies will boost progress toward the development of new therapies for diabetes.

**TABLE 1:** Insights into endocrine pancreas development and cellular identity maintenance from scRNA-Seq studies.

Key questions	*In vitro*	*In vivo*
First wave endocrine differentiation	– Human α-cells (SC-α) arise prematurely from *NKX6-1-/NEUROG3+* progenitors during Stage 4 – SC-α cells can be efficiently enriched and matured *in vitro* and *in vivo*	– Mouse first wave α-cells arise from early *Pdx1+/Sox9+/Ptf1a-/Nr2f2+* MPs – Fate and function unknown
Cell fate determination	– Subpopulations of progenitors are present during differentiation – Regulation at identified bifurcation points increase efficiency of differentiation	– Subpopulations of progenitors (MPs, BPs, EPs) are present, representing subsequent developmental stages as well as cell fate biases – Progenitors can be primed for a specific cell fate choice earlier than expected (e.g., for endocrine before *Neurog3* expression)
Islet formation	– Peninsula-like structures arise during human PSC 3D differentiation	– The peninsula theory: in mice primary islets are formed by emerging endocrine cells, adjacent to epithelium, without individual cells undergoing complete EMT
Pancreatic niche	– Mesenchymal-like cells present during human endocrine differentiation	– Mesenchyme is molecularly and functionally heterogeneous during mouse development – Heterogeneity is lower in adulthood but raises in stress and disease
Polyhormonal cells	– *GCG+/INS+* immature α-cells from Stage 4	– Transiently present in human developing pancreas and in diabetes – Not confirmed yet by scRNA-Seq
	– *TPH1+/INS^*low*^/GCG-*enterochromaffin-like cells (SC-EC) from Stage 5	– Not observed in developing/adult human pancreas
Functional maturation	– Transcriptomic changes correlate with functional maturation	– Transcriptomic changes correlate with age
Progenitors in adulthood	– *LIFR+* SC-β cell subpopulation re-enters cell cycle upon LIF exposure – Mouse *Procr+* cells can be expanded *ex vivo*	– *LIFR+* β-cell subpopulation capable of re-entering cell cycle present in mice and human – Multipotent progenitors present in mouse endocrine compartment (*Procr+* cells) and human ducts (*PDX1+*/*ALK3+*/*CAII- cells*)
Adult cells plasticity	– Dedifferentiation can be pharmacologically induced *in vitro* and *ex vivo*	– Adult endocrine and exocrine cells can dedifferentiate upon chronic stress (e.g., inflammation), upon pharmacological induction and in disease
Specificity of endocrine differentiation	– Fine-tuning of specific endocrine cell type derivation through signaling regulation at identified bifurcation points (see Figure 2)	

## Author Contributions

All authors analyzed literature, discussed findings, and wrote part of the manuscript. MB and WS did final editing.

## Conflict of Interest

The authors declare that the research was conducted in the absence of any commercial or financial relationships that could be construed as a potential conflict of interest.

## Supplementary Material

The Supplementary Material for this article can be found online at: https://www.frontiersin.org/articles/10.3389/fcell.2021.629212/full#supplementary-material. The following scRNA-Seq studies are not discussed in the manuscript, but are included in Supplementary Tables: (Grün et al., 2016; Wollny et al., 2016; Stanescu et al., 2017; Wang J. et al., 2020).
