# Supplementary material for: Endocrine Pancreas Development and Dysfunction Through the Lens of Single-Cell RNA-Sequencing
Source: Front Cell Dev Biol. 2021 Apr 29;9:629212. doi: 10.3389/fcell.2021.629212 (PMC8116659; doi:10.3389/fcell.2021.629212)
Supplement: Supplementary file 1 [file Table_1.DOCX]

**Table 1. scRNA-Seq studies on pancreas development**

| Study | Species | Model | | Developmental stage | Number of cells (QC passed) | Depth of sequencing (per cell) | | Platform | | Accession number |
| --- | --- | --- | --- | --- | --- | --- | --- | --- | --- | --- |
| **Studies in developing pancreas** | | | | | | | | | | |
| **Stanescu et al., 2017** | Mouse | wild-type (CD1 strain) | | E13.5 | 81 | 6 million reads (average) | | Fluidigm C1 | | GSE78510 |
| Main findings: | - identification of *Slc38a5* as a novel α-cell specification marker | | | | | | | | | |
| **Zeng et al., 2017** | Mouse | *mIns1-H2B-mCherry* | | P1,  P7, P14, P21, P28 | 387 | 4.3 million reads (average) | | Fluidigm C1 | | GSE86479 |
| Main findings: | - β-cell heterogeneity and transcriptomic dynamics during postnatal maturation - postnatal β-cell proliferation regulation by amino acids availability, ROS levels, and *Srf* transcription factor | | | | | | | | | |
| **Qiu et al., 2017** | Mouse | *Ins1-RFP Gcg-Cre; Rosa-RFP Ngn3-GFP* | | E17.5 P0, P3, P9, P15, P18 and P60 | 553 β-cells  338 α-cells | 1.4 million unique reads (average) | | Smart-seq2 | | GSE87375 |
| Main findings: | - β- and α-cell maturation pathways and differences between them - proliferation and maturation of β-cells are synchronized - heterogeneity of β-cells during development reflects distinct maturation states - relative transcriptional homogeneity of adult β-cells | | | | | | | | | |
| **Sznurkowska et al., 2018** | Mouse | *Rosa26-CreERT2; Rosa26-Confetti* | | E13.25  E15.25 | 516 | > 200,000 reads | | Smart-seq2 | | GSE89798 |
| Main findings: | - lineage-tracing: branching morphogenesis during second transition is driven by multipotent self-renewal progenitors at ductal ends - scRNA-Seq: molecular heterogeneity of pancreatic precursors - scRNA-Seq: pseudotime ordering suggests multipotent cells presence within trunk/ductal cluster - rare and putatively multipotent *Cpa-high/Sox9-high* cells are present at E13.25 ductal termini | | | | | | | | | |
| **Scavuzzo et al., 2018** | Mouse | wild-type (ICR strain) | | E14.5, E16.5 | E14.5 15,228 E16.5 2,006 | ~2,950 UMIs (average) | | Drop-Seq | | GSE100622 |
| Main findings: | - four EP subtypes representing subsequent maturation stages - temporal heterogeneity of BPs and EPs, including distinct endocrine lineage bias (E14.5 α fate, E16.5 β fate) - ATAC-Seq: E14.5 vs E16.5 EP chromatin accessibility shift reflecting the endocrine lineage bias | | | | | | | | | |
| **Byrnes et al., 2018** | Mouse | wild-type (C57BL/6J)  *Fev-Cre; ROSA26^mTmG^* | | E12.5, E14.5, E17.5 | E12.5 17,828 E14.5 18,224 E17.5 22,068 | ~3,100-10,250 UMIs (median) | | 10x Chromium | | GSE101099 |
| Main findings: | - *Fev+* late EPs subpopulation and endocrine lineage biases at this stage - mesenchymal heterogeneity and temporal shifts - candidate genes for epithelial–mesenchymal interactions regulation | | | | | | | | | |
| **Krentz et al., 2018** | Mouse  Human | *Neurog3-Cre; Rosa26^mTmG^*  hESC (CyT49 *NEUROG3-2A-eGFP*) | | E15.5  E18.5  *NEUROG3+* progenitors | E15.5 6,905  E18.5 6,626  4,462 | >50,000 reads | | 10x Chromium | | GSE120522 |
| Main findings: | - heterogeneity of cell fate decisions revealed by RNA velocity pseudotime analyis - candidate regulators of cell cycle exit during endocrine specification, including *Btg2* and *Gadd45a*, previously known in developing brain - identification of misdifferentiated populations during *in vitro* β-cell derivation from hESCs - comparison of hESC-derived pancreatic populations to mouse developing pancreatic cells and adult human islets | | | | | | | | | |
| **Liu et al., 2019** | Mouse | *Ngn3^eGFP/+^* | | E14.5 | 3,368 | ~56,000 reads (average) / ~5,900 UMIs (average) | | CEL-Seq | | GSE118122  GSE117616  GSE118120  GSE119110 |
| Main findings: | - transcriptomic heterogeneity of *Neurog3+* cells, which includes epigenetic factors - *Myt1+ Neurog3+* progenitors are biased towards β-cell lineage due to increased Arx enhancer methylation - DNA methylation inhibition in pancreatic progenitors promotes α-cell fate | | | | | | | | | |
| **Sharon et al., 2019b** | Mouse | *Ngn3-eGFP^+/-^* | | E13.5  E14.5  E15.5 | 440 | n/d | | Smart-seq | | GSE121416  GSE121679 |
| Main findings: | - pseudotime ordering of endocrine cells specification and corresponding temporal gene expression programs - the “peninsula” hypothesis on islet formation - hESC-derived EPs form peninsula-like buds during endocrine specification | | | | | | | | | |
| **Yu et al., 2019** | Mouse | *Pdx1-GFP*  *Pdx1-Cre; Rosa-RFP*  *Ngn3-GFP*  *Ngn3-Cre; Rosa-RFP*  *Sox9-CreER; Rosa-RFP*  *Ptf1a-CreER*  *Gcg‐P2A‐GFP*  *Ins1-RFP* | | E9.5-E17.5 daily | 144-441 per stage (Smart-Seq2)  10,904 (E14.5 10x Chromium) | ~1.3 million unique reads (average, Smart-seq2);  ~9,300 UMIs (average, 10x Chromium) | | Smart-seq2  10x Chromium | | GSE115931 |
| Main findings: | - roadmap from MPs to endocrine lineages along with identification of key branching points - early MPs as immediate ancestors of 1st wave α-cell endocrine lineage - tip-like cells with MP characteristics - MAPK/ERK regulation of endocrine progenitor specification - four subsequent EP subpopulations | | | | | | | | | |
| **van Gurp et al., 2019** | Mouse | *MIP-GFP* | | E12.5-E15.5 daily  E18.5 | 2,589 | ~17,400 UMIs (median) | | SORT-seq | | GSE132364 |
|  | - heterogeneity of epithelial tip cells corresponds to proliferation - two EP subpopulations: one marked by *Neurog3* and the second, intermediate between *Neurog3+* and endocrine cells, by *Fev* - heterogeneity of α- and β-cells reflects maturation state - pseudotime trajectories of known and novel markers along endocrine specification | | | | | | | | | |
| **Bastidas-Ponce et al., 2019** | Mouse | *Ngn3-Venus* | | E12.5-E15.5 daily | E12.5 10,790  E13.5 5,042  E14.5 9,633  E15.5 10,886 | n/d | | 10x Chromium | | GSE132188 |
| Main findings: | - *Ngn3-low* progenitors within MPCs, tip, trunk and ductal clusters - EP subpopulations-enriched and -specific marker genes and pathways - temporal heterogeneity of EPs and endocrine cells, temporal EP fate decision biases - lineage-tracing and RNA velocity analysis confirming *Fev+* EPs as direct ancestors of endocrine cells | | | | | | | | | |
| **Bakhti et al., 2019** | Mouse  Human | | datasets reanalysis (mouse E13.5-E15.5 - Bastidas-Ponce et al., 2019; human ESC pancreatic differentiation - Veres et al., 2019) | | | | | | | |
|  | - apical-basal (AB) polarity genes are downregulated, while adherens and tight junctions (AJs/TJs) genes undergo dynamic changes during endocrine specification at the *Ngn3*-high and *Fev+* stages, reflecting delamination process - similar dynamic expression patterns are present in hPSC-derived pancreatic progenitors during endocrine fate induction - mouse and human 3D polarized pancreatic epithelial cysts culture system for studies on endocrine fate commitment | | | | | | | | | |
| **Wang et al., 2020b** | Mouse  Human | | datasets reanalysis (Petersen et al., 2017, Byrnes et al., 2018; Qiu et al., 2017; and Wollny et al., 2016) | | | | | | | |
| Main findings: | - a mathematical model of core endogenous network of pancreatic progenitors, which predicts novel pancreatic progenitor types, and is validated by comparison with single-cell data | | | | | | | | | |
| **Studies in differentiated hPSCs** | | | | | | | | | | |
| **Balboa et al., 2018** | Human | | patient-derived INS C96R iPSC line and mutation-corrected isogenic line | 3D β-cell differentiation: islet-like stage | 2,171 | ~3300 UMIs (average) | inDrops | | GSE115257 | |
| Main findings  : | - heterozygous proinsulin misfolding (C96R) mutation, which cause neonatal diabetes, induce ER-stress in iPSC-derived cells committed to β-cell fate - the ER-stress results in UPR activation, mitochondrial changes, impaired mTORC1 signaling, proliferation and maturation of β-cells *in vitro* and upon transplantation | | | | | | | | | |
| **Veres et al., 2019** | Human | | ESCs (HUES8 line)  iPSCs (mixed iPS 1016 and iPS 1031 lines) | 3D β-cell differentiation: stages 3-6 (14 timepoints) | stages 3-6 - >46,000 (various protocols & cell lines)  stage 5 time course - 51,274  stage 6 time course - 38,494 | >1000 UMIs  >750 UMIs (stages 5 & 6 time courses) | inDrops | | GSE114412 | |
| Main findings: | - in depth, time-lapse roadmap of hPSC-to-SC-β differentiation and maturation process revealing misdifferentiation events - precocious differentiation of *PDX1+ NKX6.1-* progenitors into endocrine lineage during stage 4 leads mainly to polyhormonal α-like cells - endocrine lineages specified during stage 5 from NKX6.1+ progenitors develop with similar rates into SC-β or enterochromaffin-like, serotonin-producing *TPH1+* cells (SC-ECs) - high *CD49a* expression distinguish SC-β cells from other endocrine lineages - extended stage 6 culture (several weeks) promotes SC-β maturation - a significant portion of non-endocrine (ductal, acinar and mesenchymal) cells is produced, and can be removed by stage 6 re-aggregation | | | | | | | | | |
| **Sharon et al., 2019a** | Human | | ESCs (HUES8 line) | 3D β-cell differentiation: stages 1-6 (10 timepoints) | 720 | 0.9 million reads (median) | Fluidigm C1 | | NCBI SRA: PRJNA532884 | |
| Main findings: | - differentiation process is relatively homogenous up to pancreatic progenitor stage (stages 1-3) - endocrine specification occurs in two waves, during stage 4 (leading to polyhormonal α-like cells) and stage 5 (SC-β) - BMP pathway stimulates progenitors proliferation (stage 4), while Wnt pathway inhibits endocrine specification (Wnt inhibition during stages 4 and 5 can increase endocrine cells yield by 50%) | | | | | | | | | |
| **Rosardo-Olivieri et al., 2020** | Human | | YAPS6A-overexpressing and ctrl ESCs (HUES8 line) | 3D β-cell differentiation: SC-β stage | 11,517 | n/d | 10x Chromium | | GSE137961 | |
|  | - molecular characterization of SC-β cells induced to proliferate by constitutively active YAP (YAPS6A), a Hippo effector - LIF induces proliferation, via JAK/STAT and CEBPD pathways, in a LIFR+ β-cells subset *in vivo* and *in vitro* | | | | | | | | | |
| **Hogrebe et al., 2020** | Human | | ESCs (HUES8 line) | mixed 3D/2D β-cell differentiation: stage 4 (PP2) | 1,062 | n/d | 10X Chromium | | GSE137659 | |
| Main findings: | - cytoskeletal state regulates pancreatic differentiation - F-actin depolymerization by Lantrunculin A promotes endocrine specification of pancreatic progenitors, while cytoskeleton hyperactivation by Nocodazole drives exocrine specification - F-actin depolymerization at the EP stage (beginning of stage 5) enables production of functional SC-β cells competent to rescue diabetic mice with the planar differentiation protocol | | | | | | | | | |
| **Maxwell et al., 2020** | Human | | Wolfram Syndrome patient-derived iPSC line (WS4) and a mutation-corrected isogenic line (WS4 corr) | 3D β-cell differentiation: stage 6 (SC-β) | 7,215 WS4,  3,640 WS4 corr | 50,996 reads (average) | 10X Chromium | | GSE139535 | |
| Main findings: | - iPSCs from Wolfram Syndrome (WS) patients fail to efficiently differentiate into SC-β cells and other endocrine lineages, in favour of exocrine and non-pancreatic lineages - genetic corrections of causative mutations in *WFS1* gene rescue the phenotype - WS SC-β cells have increased ER- and mitochondrial stress and apoptotic markers as compared to corrected lines | | | | | | | | | |
| **Peterson et al., 2020** | Human | | ESCs (HUES8 line) | 3D α-cell differentiation: stage 5 (inDrops) | 2,043 (inDrops) | n/d | inDrops  10X Chromium | | GSE138857 | |
| Main findings: | - a modified differentiation protocol that induces precocious (prior to NKX6-1 expression) endocrine lineage commitment of pancreatic progenitors efficiently give rise to α-cell lineage - polyhormonal pre-α-cells (stage 5) upon protein kinase C (PKC) activation undergo transcriptomically subtle maturation into functional, monohormonal SC-α cells | | | | | | | | | |
| **Russel et al., 2020** | Human | | *INS*^WT/GFP^ iCas9 hESCs - *MAFB*+/+ and *MAFB*-/- | 3D β-cell differentiation: PP and β-like stages | PP:  578 MAFB+/+ 564 MAFB−/−  β-like:  1,126 MAFB+/+  2,387 MAFB−/− | n/d | 10X Chromium | | GSE145347 | |
| Main findings: | - MAFB loss limits β- and α-cell specification and function - MAFB does not affect pancreatic progenitor specification and identity - MAFB-/- hESC more efficiently differentiate into *SST-*, *PPY-*, *GAST-*, and *PYY*-expressing endocrine cells | | | | | | | | | |
| **Augsornworawat et al., 2020** | Human | | ESCs (HUES8 line)  iPSCs (WS4 corr line) | 3D β-cell differentiation:  stage 6 (SC-β) and grafted cells (for 6 months in STZ-diabetic mice) | 45,674 | n/d | 10x Chromium | | GSE151117 | |
|  | - multiple endocrine cell lineages that emerged during *in vitro* differentiation persist in long-term grafts - SC-α and SC-β cells undergo maturation upon grafting, and more closely resemble their cadaveric islet counterparts - SC-EC cells upon grafting acquire new transcriptomic features and lose pancreatic endocrine markers | | | | | | | | | |
