# Supplementary material for: Endocrine Pancreas Development and Dysfunction Through the Lens of Single-Cell RNA-Sequencing
Source: Front Cell Dev Biol. 2021 Apr 29;9:629212. doi: 10.3389/fcell.2021.629212 (PMC8116659; doi:10.3389/fcell.2021.629212)
Supplement: Supplementary file 2 [file Table_2.DOCX]

**Table 2. scRNA-Seq studies in adult pancreas**

| Study | Species | Source (number of donors) | Number of cells  (QC passed) | Depth of sequencing (average per cell) | Platform | Accession number  (NCBI GEO) |
| --- | --- | --- | --- | --- | --- | --- |
| **Human studies** | | | | | | |
| **Li et al., 2016** | Human | ND (1) | 64 | 12.7 million reads | Smart-seq2 | GSE73727 |
| Main findings: | - Identification of novel transcription factors specific for endocrine cell types - Transcriptional repressor REST is specifically expressed in exocrine pancreas - Identification of genes with opposite expression patterns in mouse and human α- and β-cells | | | | | |
| **Xin et al., 2016** | Human | ND (12) and T2D (6) | 1,492 | 0.95 ± 0.46 million reads | Fluidigm C1 | GSE81608 |
| Main findings: | - Identification of cell type-specific genes and pathways - Identification of differentially expressed genes between healthy and T2D individuals - Majority of T2D-altered genes have unknown function in pancreas - Identification of differentially expressed genes between mouse and human α- and β-cells | | | | | |
| **Segerstolpe et al., 2016** | Human | ND (4) and T2D (2) | 2,209 | 0.75 million reads | Smart-seq2 | E-MTAB-5061 |
| Main findings: | - Transcriptional profiling of rare pancreatic cell types (δ-, PP-, ε-, and stellate cells) - hints for unknown functions - δ-cells specifically express leptin receptor (LEPR) and ghrelin receptor (GHSR) implicating role in metabolic signal coordination within islets (see also Lawlor et al., 2017, Fang et al., 2019) - Uncovering subpopulations of acinar, α- and β- cells - T2D alters gene expression in multiple endocrine and exocrine cell types - Identification of genes negatively or positively correlated with BMI - β and δ cells express GCG-like peptide receptor 1 (*GLP1R*), targeted by GLP1 analogs used as anti-diabetic drugs | | | | | |
| **Grün et al., 2016** | Human | ND (5) | 1,752 | 4,885 transcripts (median) | CEL-seq | GSE81076 |
| Main findings: | - *de novo* (StemID) prediction of novel, putative multipotent progenitor subpopulations within adult human ducts, marked by *CEACAM6* or ferritin protein components (*FTH1*, *FTL*) - *FTL+INS+* cells exist in pancreatic ducts | | | | | |
| [**Wang et al., 2016**](https://www.zotero.org/google-docs/?BEpPoq) | Human | ND (3),  T1D (1),  T2D (2); ND children (2; 19mo, 2y) | 635 | 2.2 million reads (median) | Fluidigm C1 | GSE83139 |
| Main findings: | - α-and β-cell signature in children is less-defined than in adults - T2D α- and β-cells resemble juvenile α- and β-cells (putative dedifferentiation process) - Active Sonic hedgehog pathway characterize proliferating α-cells; - Cells with mixed α-/β- or β-/ductal signature might reflect rare cell types or cell states | | | | | |
| **Baron et al., 2016** | Human  Mouse | ND (4)  wild-type (2; C57BL/6 and ICR strains) | 8,629  1,886 | 0.1 million reads (average) | inDrops | GSE84133 |
| Main findings: | - Characterization of endocrine and non-endocrine cell transcriptomes - Focus on stellate cell activation, Schwann cell dedifferentiation, ductal cells subpopulations - β-cell heterogeneity corresponds to ER-stress - bulk RNA-Seq deconvolution using scRNA-Seq data | | | | | |
| **Muraro et al., 2016** | Human | ND (4) | 4,416 | 14,604 transcripts (average) / 40,000 reads (average) | SORT-Seq | GSE85241 |
| Main findings: | - Characterization of cell-type-specific genes, transcription factors, and cell-surface markers for endocrine (α-, β-, δ-, PP-, ε-cells) and non-endocrine cells (duct, acinar, mesenchymal, endothelial) - outlier acinar and β-cell populations identified by StemID - enrichment of α- or β-cells by CD24 and TM4SF4 surface markers | | | | | |
| **Lawlor et al., 2017** | Human | ND (5) and T2D (3) | 978 | 3 million reads | Fluidigm C1 | GSE86473 |
| Main findings: | - Determination of specific markers and enriched gene sets of endocrine cells (α-, β-, δ- and PP-cells) - Attribution of of known diabetes-related genes’ expression to islet cell types - Identification of cell-type specific alterations in T2D | | | | | |
| **Enge et al., 2017** | Human | ND (8): 1 month to 54 years old | 2,544 | 3,203 transcripts (total) | Smart-seq2 | GSE81547 |
| Main findings: | - Transcriptional noise increase with age, including elevated expression of stress-related genes, increased number of polyhormonal *INS+GCG+* cells, and other signs of cell fate drift - scRNA-Seq can detect stochastic age-related mutations, attributed to oxidative-stress induced DNA lesions | | | | | |
| **Fang et al., 2019** | Human | ND (6, incl. 3 w/ BMI>30)  T2D (3, incl. 2 w/ BMI>30) | 28,026 | 2,206 UMIs (average) | Drop-Seq | GSE101207 |
| Main findings: | - transcriptional profiling of non-endocrine populations, including activated and quiescent pancreatic stellate cells - determination of endocrine-specific gene signatures for each cell type, including transcription factors, GWAS-risk genes and cell surface genes - identification of a proliferating α-cell subpopulation, and donor-specific α- and β-cell subpopulations - the trajectory-based RePACT strategy reveals α- and β-cells affected by T2D or diabetes, as well as common and distinguishing transcriptomic changes for each condition - integration of scRNA-Seq data with genome-wide CRISPR screen identified novel insulin release regulatory complexes, i.e. cohesin function and NuA4/Tip60 histone acetyltransferase complexes | | | | | |
| [**Camunas-Soler et al., 2020**](https://www.zotero.org/google-docs/?60I55M) | Human | ND (18)  T2D (7)  T1D (cryopreserved) | 1,021 (Patch-Seq) 3,518 (scRNA-Seq only)  348 (T1D) | 1-2 million reads (average) | Smart-seq2 | GSE124742 |
| Main findings: | - β-cells are electrophysiologically heterogenous - Identification of genes, pathways and networks positively or negatively correlating with β-cell size, exocytosis function and electrophysiological properties - Gene expression pattern is predictive for β-cell electrophysiological properties - *RBP4* marks β-cell subpopulation with reduced functionality - β-cells in T2D and obesity attempt to compensate for increased insulin demands by inducing pro-functional programs - Pathways involved in functional impairment in T2D β-cells are different than in healthy β-cells, involving ETV1 and STAT3 pathways - Uncovering the transcriptional and physiological heterogeneity in T1D α-cells - Patch-Seq approach can be used for both fresh and cryo-banked islets | | | | | |
| **Qadir et al., 2020** | Human | ND (3) | 6,873 (CD90-/ALK3^bright+^ cells) | 10,315-13,777 UMIs (median) | 10X Chromium | GSE131886 |
| Main findings: | - heterogeneity within epithelial compartment of major pancreatic ducts (MPDs) and pancreatic duct glands (PDGs) - identification of transitional pro-ductal or ducto-acinar progenitors within MPDs and PDGs - PDX1+/ALK3+/CAII— progenitors are multipotent, giving rise to non-endocrine and endocrine cells upon grafting - multipotent progenitors are retained in T1D and T2D pancreata regardless of the disease stage | | | | | |
| **Marquina-Sanchez et al., 2020** | Human  Mosue | ND (4)  n/d | 107,192 total islet cells | ~5,000-22,000 UMIs (median) | 10X Chromium  Drop-Seq | GSE147203  GSE147202 |
| Main findings: | - spike-in reference cells allow decontamination of scRNA-Seq data from significant cell-free RNA contamination - FoxO inhibition in mouse and human islets *in vitro* induces α- and β-cell dedifferentiation - arthemeter induce *INS* and β-cell signature in a subset of α-cells in mouse and human islets *in vitro* - arthemeter and GABA induce β-cell dedifferentiation in mouse but not in human | | | | | |
| **Avrahami Kaestner et al. 2020** | Human | ND: neonatal (1; 18d),  toddler (5; 10mo-4y), adolescent (2; 11-12y) adult (4; 21-61y);  T2D (11; 37-64y) | 619 | 1.7±1.3 million reads | Fluidigm C1 | GSE154126 |
| Main findings: | - Age-dependent β-cell transcriptomic changes - Mature α-cells retain immature transcriptomic signatures - T2D induce juvenile and non-endocrine signatures in α- and β-cells, suggesting dedifferentiation process - Identification several new genes deregulated in T2D donors | | | | | |
| **Tosti et al. 2020** | Human | newborn (2; 1d),  juvenile (1; 18mo), adult (2; 30-53y);  chronic pancreatitis (2)  pancreatic tumor (3) | 10,528 newborn  112,564 adult  2,726 chronic pancreatitis | 1,287 UMIs per nucleus (mean for all samples) | NextSeq 500 (Illumina)  sNuc-Seq  *in situ* sequencing | EGAS00001004653 |
|  | - single-nucleus RNASeq (sNuc-Seq) from snap-frozen tissues allows efficient profiling of whole pancreas, including exocrine compartment, without RNA degradation by nucleases - uncovered three different acinar cell types:   acinar-s - typical acinar cells with higher expression of digestive enzymes;  acinar-i - plastic population responsive to external stimuli (e.g. islet signals), with lower expression of digestive enzymes; acinar-REG+ - linked to pancreatic lesion development;   - comparison of neonatal and adult pancreas showed different endocrine cell composition - more endocrine in neonatal than in adult; no acinar-REG+ population - identification of cell clusters specific for chronic pancreatitis (tuft cells, mast cells, monocytes, enriched REG+ cells) as compared to healthy pancreas | | | | | |
| **Mouse studies** | | | | | | |
| **Wollny et al., 2017** | Mouse | *H2B-mCherry* strain | 108 (acinar) | 5 millions reads (average) | Smart-seq2 | GSE80032 |
| Main findings: | - proliferative acinar cells heterogeneity revealed by *in vitro* organoid formation and *in vivo* lineage-tracing - scRNA-Seq revealed rare (1%) *Stmn1+* acinar-like progenitors - STMN1+ acinar subpopulation is present in human pancreas - *Stmn1+* marks proliferation-capable progenitors and is transiently induced upon injury in larger fraction of cells, suggesting acinar cell plasticity | | | | | |
| **Lu et al., 2018** | Mouse | wild-type (control and high-fat diet)  βEedKO mice  Min6-B1 cell line | 300  n/d  359 | ~15,000 unique reads | CEL-Seq2 | GSE110648 |
| Main findings: | - integration of scRNA-Seq data with bulk β-cell epigenome profiling revealed chromatin-state-defined transcriptome dysregulation and dedifferentiation in high-fat diet mice - the epigenetic changes result from polycomb repressor complex 2 (PRC2) dysfunction, and are observed in T2D patients, too - β-cell identity is maintained by PRC2 function - ectopic expression of PRC2-silenced transcription factors enriched in T2D triggers β-cell dedifferentiation *in vitro* and *in vivo* - epigenetic targeting might be considered as a therapeutic option for T2D | | | | | |
| **Wang et al., 2020a** | Mouse | wild-type (ICR strain)  *Procr+* organoids (7d, 28d, grafted) | 7,160 (tissue)  5,025 (organoids)  2,129 (grafted) | ~3,000- 50,000 UMIs | 10x Chromium | biosino.org:  OEP000249  OEP000250 |
| Main findings: | - scRNA-Seq revealed *Procr+* endocrine progenitors in adult mice - *Procr+* cells are enriched in cell migration, epithelial-to-mesenchymal transition, and Wnt signaling genes - *Procr+* cells give rise to new β, α, δ, and PP cells in homeostasis as assessed by lineage-tracing - When co-cultured with endothelial cells, *Procr+* cells can form functional islet-like organoids *in vitro* - *Procr+* *in vitro* organoids can expand for long-term and reverse diabetes when grafted | | | | | |
| **Sachs et al., 2020** | Mouse | healthy,  mSTZ-treated  mSTZ+oestrogen  mSTZ+GLP-1, mSTZ+GLP-1-oestrogen, mSTZ+PEG-insulin  mSTZ+GLP-1-oestrogen+ PEG-insulin;  (C57BL6J strain, n=3 for each condition) | 7,578 5,001  4,889  3,874  5,201  3,217 3,276 | n/d | 10x Chromium | GSE128565 |
|  | - Mature and immature and/or proliferative β-cells are present in adult mice - Surviving β-cells in mSTZ mice resemble immature, embryonic β-cells - Mechanisms of β-cell regeneration and redifferentiation upon chronic PEG-insulin and GLP-1-oestrogen treatments, separately and in combination, in mSTZ mice | | | | | |

ND, non-diabetic; T1D, type 1 diabetes; T2D, type 2 diabetes; Patch-seq, combined whole-cell patch-clamp measurements and scRNA-seq; mSTZ, multiple-low-dose model of streptozotocin-induced diabetes; GLP-1, Glucagon-like peptide-1; PEG-insulin, pegylated insulin analogue; REST, RE1-Silencing Transcription factor
